# Obesity-Related Microenvironment Promotes Emergence of Virulent Influenza Virus Strains

**DOI:** 10.1128/mBio.03341-19

**Published:** 2020-03-03

**Authors:** Rebekah Honce, Erik A. Karlsson, Nicholas Wohlgemuth, Leonardo D. Estrada, Victoria A. Meliopoulos, Jiangwei Yao, Stacey Schultz-Cherry

**Affiliations:** a Department of Infectious Diseases, St. Jude Children’s Research Hospital, Memphis, Tennessee, USA; b Integrated Biomedical Sciences Program, University of Tennessee Health Science Center, Memphis, Tennessee, USA; Columbia University Medical College

**Keywords:** NHBE, influenza, interferons, obesity, viral evolution, virulence

## Abstract

Obesity is associated with increased disease severity, elevated viral titers in exhaled breath, and significantly prolonged viral shed during influenza A virus infection. Due to the mutable nature of RNA viruses, we questioned whether obesity could also influence influenza virus population diversity. Here, we show that minor variants rapidly emerge in obese mice. The variants exhibit increased viral replication, resulting in enhanced virulence in wild-type mice. The increased diversity of the viral population correlated with decreased type I interferon responses, and treatment of obese mice with recombinant interferon reduced viral diversity, suggesting that the delayed antiviral response exhibited in obesity permits the emergence of a more virulent influenza virus population. This is not unique to obese mice. Obesity-derived normal human bronchial epithelial (NHBE) cells also showed decreased interferon responses and increased viral replication, suggesting that viral diversity also was impacted in this increasing population.

## Abstract (precis)

Currently, 50% of the adult population worldwide is overweight or obese. In these studies, we demonstrate that obesity not only enhances the severity of influenza infection but also impacts viral diversity. The altered microenvironment associated with obesity supports a more diverse viral quasispecies and affords the emergence of potentially pathogenic variants capable of inducing greater disease severity in lean hosts. This is likely due to the impaired interferon response, which is seen in both obese mice and obesity-derived human bronchial epithelial cells, suggesting that obesity, aside from its impact on influenza virus pathogenesis, permits the stochastic accumulation of potentially pathogenic viral variants, raising concerns about its public health impact as the prevalence of obesity continues to rise.

## INTRODUCTION

The obesity epidemic is an ever-expanding threat to public health. The World Health Organization estimates over 50% of the current global population is overweight or obese, with prevalence increasing annually (1, 2). The obese host provides a unique microenvironment for disease pathogenesis, characterized by a state of chronic, low-grade inflammation, resulting in suppressed innate and adaptive immune responses (1, 3, 4). While early studies suggested an increase in influenza virus pathogenicity, obesity was epidemiologically linked to increased risk of severe disease in human populations during the 2009 influenza A virus (IAV) H1N1 pandemic (5–8). Indeed, in IAV-infected individuals, a body mass index (BMI) greater than 30 is associated with increased hospitalizations, secondary infections, and mortality (9, 10). Subsequently, this association has been shown globally, and obese individuals are now considered at high risk for serious complications from influenza infection (11–13).

Understanding the impact of obesity on the immune response to IAV has been accomplished using genetically obese (OB) mice, namely, *ob/ob* mice (strain B6.Cg-*Lep^ob^*/J) lacking the leptin signaling molecule, and diet-induced models of obesity (DIO). In DIO mice, IAV-infected mice had seven times greater mortality (7) and lung pathology (14), as well as delayed wound repair (15), compared to lean controls. Similarly, studies on IAV infection in OB mice show increased disease severity (16), increased risk of secondary bacterial infections (17), reduced vaccine efficacy, and impaired wound healing (18). Increased IAV disease severity may be due to a delayed and blunted immune response, because the interferon (IFN) (7) and adaptive cellular (8, 15) and antibody-mediated (19) responses all are reduced in obese mice. These findings are corroborated by human cohort studies, in which obese humans fail to induce robust T-cell and antibody-mediated immunity upon infection and vaccination and shed higher quantities of infectious virus longer than lean subjects (20–23).

RNA viruses like IAV replicate using an RNA-dependent RNA polymerase (RdRp) that lacks proofreading functionality, resulting in a high error rate during viral replication and emergence of minor variants that may affect viral fitness (24–26). Interestingly, host status is implicated in impacting acute, within-host viral evolution. Distinct from their impacts on host immunity, dietary oxidative stress (27), levels of vitamins and minerals (28, 29), and aging (30) can increase the virulence of a viral population.

Determining obesity’s impact on within-host viral evolution is of paramount concern in an increasingly obese world. Based on the negative effects obesity exerts on the host immune response and the knowledge that host nutritional status increases the emergence of potentially pathogenic viral variants, we hypothesized that passaging IAV through the obesogenic lung microenvironment would alter viral evolution compared to virus passaged through a lean microenvironment. The unique immune status in the obese host could impact the viral population itself, leading to the generation of novel, minor viral variants with increased virulence. We found that serial passaging of a human H1N1 influenza virus through DIO or OB mice results in a more virulent IAV population versus passage through a lean host. Obese host-passaged viruses replicated to higher viral titers and induced increased morbidity in wild-type (WT) C57BL/6 mice. These findings are not limited by viral subtype, because passaging a human H3N2 seasonal virus that has no discernible pathogenicity in WT mice through obese mice generated a strain that could productively infect and cause disease in WT mice. Deep-sequencing viruses derived from obese- and lean-host viruses revealed several mutations in obese host-passaged viruses associated with virulence in the mouse model. Strikingly, this evolution phenotype was retained using human primary respiratory cells. IAV replicated to higher titers in normal human bronchial epithelial cells (NHBE) from obese donors than in those from lean individuals. Obese-donor NHBE-derived virus displayed increased replication kinetics and cell death in Madin-Darby canine kidney (MDCK) cells. Both obese mice and obese-donor NHBEs exhibited a reduction in interferon and interferon-stimulated genes (ISGs) produced postinfection (p.i.), potentially reducing the ability to control viral spread and limit viral replication (31, 32). Further, the increased diversity and virulence was sensitive to host interferon responses, because exogenous treatment of OB mice with interferon reduced viral diversity. Overall, we conclude that obesity results in a unique microenvironment characterized by an impairment of the interferon response that is permissive for the generation of more virulent IAV populations and faster viral host adaptation.

## RESULTS

### Experimental evolution of H1N1 influenza virus in obese mice results in increased disease severity.

**FIG 1:**
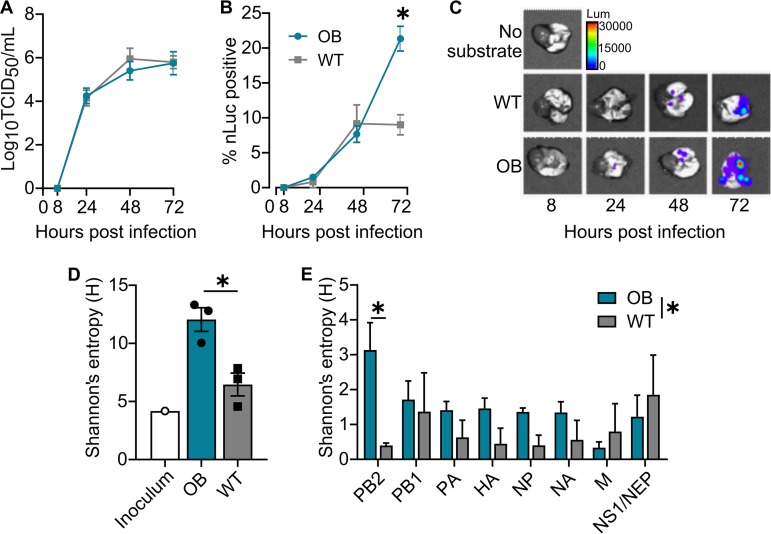
Influenza infection of obese hosts results in greater viral spread and produces a more diverse viral quasispecies. (A to C) OB and WT mice inoculated with a bioluminescent reporter CA/09-nLuc virus (*n* = 5 mice/genotype/time point). (A) No difference in viral titers recovered from lungs from day 0 to 3 p.i. (B) Bioluminescent flux measured in excised lungs shows increased viral spread at day 3 p.i. in OB mice (*n* = 5 mice/time point). Results were analyzed by two-way analysis of variance (ANOVA) with mouse genotype as the source of variation (*P = *0.0186, *F* = 6.106). (C) Representative images of viral spread. Data are representative of 3 independent experiments as described for panel B with *n* = 3 to 5 mice/experiment. (D and E) Shannon's entropy of the viral population is increased in OB-derived viruses at day 3 p.i. (D) Diversity measures of all genome segments; unpaired *t* test for OB versus WT, *P = *0.0167, *t* = 3.959, df = 4. (E) Diversity measures by genome segment, two-way ANOVA, and source of variation in diversity for mouse genotype (*P = *0.0260; segment PB2, *P = *0.0203). Data represented in panels A, B, D, and E are means ± standard errors (*, *P < *0.05).

Using an A/California/04/2009 (CA/09) H1N1 luciferase reporter virus (33, 34), we observed that genetically obese (OB) mice, despite statistically similar viral titers (Fig. 1A), had increased viral spread in the lungs compared to that of C57BL/6 (WT) mice (*P* < 0.0001) (Fig. 1B and C) at day 3  postinfection (p.i.) (17). To determine if the enhanced viral spread was associated with changes in the viral genome, viral RNA was extracted from lung homogenates, amplified using universal influenza primers, and deep sequenced (17). While no consensus changes were noted, analysis of single-nucleotide variants (SNVs) at a frequency of greater than 5% in the viral population showed greater mean within-host diversity in the lungs of obese mice than the wild type, as measured via Shannon**’**s entropy (*H*) (*P* = 0.02) (Fig. 1D). Although there was a trend for increased diversity in several genes, only the level of the PB2 gene was significantly increased in obese mice (*P* = 0.02) (Fig. 1E).

**FIG 2:**
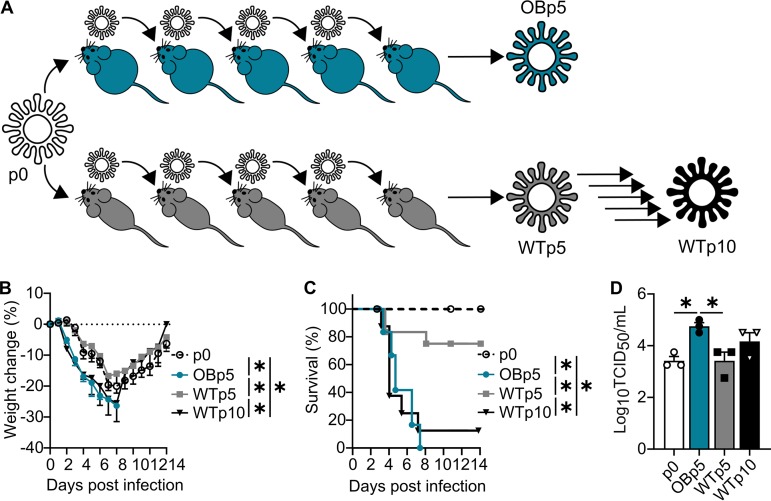
Obese host-derived viruses exhibit greater virulence upon infection of a wild-type host. (A) CA/09 virus experimental evolution scheme in OB and WT mice. (B) Percent weight change of WT mice inoculated with 300 TCID_50_ of the indicated viruses. (C) Percent survival of WT mice inoculated with 300 TCID_50_ of the indicated viruses. (D) Lung viral titers recovered at day 3 p.i. from *n* = 3 to 5 WT mice inoculated with the indicated viruses. Data were analyzed for panel B with ordinary two-way ANOVA with Tukey’s multiple-comparison test up to day 8 p.i., for panel C with Mantel-Cox log rank test, and for panel D with ordinary one-way ANOVA with Tukey’s multiple-comparison test. Data represented are means ± standard errors (*, *P < *0.05). See also Fig. S1.

This finding led us to question whether the obesogenic microenvironment could impact within-host viral evolution of IAV. Thus, OB and WT mice were intranasally inoculated with 10^3^ 50% tissue culture infectious doses (TCID_50_) of A/California/04/2009 (CA/09) H1N1 virus. Lungs were collected day 3 postinfection (p.i.) and homogenized, and viral titers were determined. Naïve OB and WT mice were then intranasally inoculated with 10^3^ TCID_50_ of diluted, pooled lung homogenate for a total of 5 or 10 passages, respectively (Fig. 2A). This scheme was performed twice, and results reported are representative of the two independent experiments.

10.1128/mBio.03341-19.1 FIG S1 Experimental evolution of influenza virus in obese mice results in increased disease severity. Influenza A virus A/California/04/2009 (pdmH1N1) experimental evolution through *n* = 3 to 5 WT mice per passage for 10 passages or OB mice for 5 passages. (A) Percent weight change from baseline at day 3 p.i. in OB and WT mice. (B) Viral titer recovered at day 3 p.i. from lungs of *n* = 5 WT or OB mice. (C) Clinical scores of OB and WT at day 3 p.i. Data were analyzed with ordinary two-way ANOVA with Sidak’s correction. Data are presented as means ± SEM (*, *P* < 0.05). Data are representative of two independent experiments. Download FIG S1, JPG file, 0.6 MB. Copyright © 2020 Honce et al. 2020 Honce et al. This content is distributed under the terms of the Creative Commons Attribution 4.0 International license.

No statistical differences in weight loss or viral titer were observed (see Fig. S1A and B in the supplemental material); however, OB mice displayed increased clinical scores and disease severity at viral passage 3 (*P = *0.04), 4 (*P = *0.0015), and 5 (*P < *0.0001) compared to WT controls, with increased severity during passaging observed only in OB mice (Fig. S1C). Because of this, passaging through the obese model was stopped after passage 5 for humane reasons.

Due to the increased clinical severity in obese models following passaging, we next questioned whether obese host-passaged viruses would induce greater morbidity and mortality in WT mice. We inoculated WT mice with increasing doses of the original CA/09 viral stock (p0), the virus passaged five times through OB mice (OBp5), or the virus passaged five or ten times through WT mice (WTp5 or WTp10, respectively) and monitored for mortality (Fig. 2A). The 50% mean lethal dose (MLD_50_) was decreased with OBp5 virus (10^1.92^ TCID_50_/ml) versus p0 virus (10^3.92^ TCID_50_/ml) and WTp5 virus (10^3.66^ TCID_50_/ml), indicating that passaging CA/09 through obese animals led to increased virulence. To test if increased morbidity was associated with severity and viral titer, WT mice were inoculated with 300 TCID_50_/ml of p0, OBp5, WTp5, or WTp10 virus and monitored for 14 days. WT mice inoculated with OBp5 virus had significantly increased weight loss up to day 8 p.i. (versus p0, *P < *0.001; versus WTp5, *P < *0.001) (Fig. 2B) and mortality (*P < *0.0001) (Fig. 2C) compared to WT mice inoculated with p0 or WTp5 virus; however, these differences were mitigated with WTp10 virus (35). During the generation of the WTp10 virus, WT mice did not suffer increased disease severity, yet upon infection with WTp10 virus, we observed reduced survival compared to that of p0 virus. We attribute these differences to increased disease severity upon infection with WTp10 after day 3 p.i., which was our collection time point for the generation of the WTp10 virus. Increased morbidity and mortality due to the OBp5 virus were associated with increased lung viral titers at day 3 p.i. (versus p0, *P = *0.03; versus WTp5, *P = *0.03) (Fig. 2D).

### Emergence of a virulent phenotype is not specific to obesity model or viral strain.

To determine whether the observed phenotype was unique to genetically obese mice, we repeated the studies using the diet-induced obese (DIO) model. Similar results were observed in DIO mice and with mice fed a low-fat diet or regular chow (LN) for a similar time as controls. Weight loss was similar between DIO and LN mice until passage 5, at which point DIO mice lost significantly more weight than LN by day 3 p.i. (*P = *0.0001) (Fig. S2A). Viral titers at day 3 p.i. were similar between the DIO and LN models at each passage except for passage 2, when increased viral titers were recovered from DIO mice (*P = *0.04) (Fig. S2B). Like OB mice, DIO mice had significantly increased clinical scores at virus passages 4 (*P = *0.02) and 5 (*P = *0.0012) (Fig. S2C). Upon inoculation of WT mice, DIOp5 virus induced increased weight loss, although not at a statistically significant level (Fig. S2D), and mortality (*P = *0.04) (Fig. S2E) in WT mice compared to LNp5 virus-inoculated mice. Lung viral titers were 10× higher in DIOp5-inoculated mice than in mice inoculated with p0 (*P = *0.0001) and LNp5 (*P < *0.0001), and the MLD_50_ was 10^2.66^ TCID_50_/ml for DIOp5 and >10^3.00^ TCID_50_/ml for LNp5 (Fig. S2F).

10.1128/mBio.03341-19.2 FIG S2 Emergence of a virulent phenotype is not specific to obesity model. (A to C) Serial passage of pdmH1N1 through *n* = 3 to 5 DIO and LN mice. Shown are weight changes (A), lung viral titers (B), and clinical disease scores (C) measured at day 3 p.i. for DIO and LN mice at each passage. (D to F) WT mice (*n* = 5 mice/virus) inoculated with CA/09 virus passaged 5 times through DIO mice (*n* = 3 to 5 mice/passage) showed increased weight loss (D) and reduced survival (E) compared to those of virus passaged through LN mice or the parental viral strain p0. (F) Viral titers at day 3 p.i. are increased in WT mice inoculated with DIO-derived virus compared to those of LN-derived and parental viruses (*n* = 3/virus). (G) MDCK cells (*n* = 3 wells/virus) inoculated at a multiplicity of infection (MOI) of 1.0 for single-cycle replication kinetics of p0, DIOp5, and LNp5 viruses. (H) MDCK cells (*n* = 3 wells/virus/time point) inoculated at an MOI of 0.01 with DIOp5 virus shows increased replication kinetics compared to those of inoculation with p0 and LNp5 viruses. (I) Shannon’s entropy is increased in DIO-derived viruses (*n* = 6 viruses/genotype from passage 1 and passage 5) compared to those of LN-derived viruses. Data were analyzed for panels A to C and F with ordinary one-way ANOVA, for panels D, G, and H with ordinary two-way ANOVA with Sidak’s multiple-comparison test, for panel E with Mantel-Cox log-rank, and for panel I with unpaired *t* test. Data are represented as means ± standard errors (*, *P* < 0.05). Download FIG S2, JPG file, 1.4 MB. Copyright © 2020 Honce et al. 2020 Honce et al. This content is distributed under the terms of the Creative Commons Attribution 4.0 International license.

A potential practical application of our findings is the ability to quickly adapt human influenza viruses to mice. Contemporary (post-2010) H3N2 viruses have been difficult to study in laboratory settings due to poor *in vitro* and *in vivo* replication. It is hard to discern viral titers, because H3N2 viruses poorly hemagglutinate turkey and chicken red blood cells (36–38). We questioned whether OB mice could be used to rapidly adapt these viruses for use *in vivo* or if our findings were unique to CA/09 H1N1 virus. OB and WT mice were inoculated with A/Switzerland/9715293/2013 (H3N2) virus as described above. Viral titers steadily and significantly increased over 5 blind passages in OB mice (*P = *0.008) (Fig. S3A). Like CA/09 virus, the OB-derived H3N2 virus induced significantly increased weight loss (*P < *0.0001) (Fig. S3B) and viral titers (*P = *0.003) (Fig. S3C) in WT mice compared to those in the parental strain. In contrast, we were unable to recover any virus from WT mice at day 3 p.i. in the initial infection, so passaging through the WT background could not be completed. These results highlight that the obese mouse supports the replication of seasonal H3N2 viruses and can be used to rapidly adapt viruses for *in vivo* study. Taken together, obesity promotes the evolution of an influenza virus population with increased virulence, even upon inoculation of a WT host.

10.1128/mBio.03341-19.3 FIG S3 Emergence of a virulent phenotype is independent of viral strain. (a) H3N2 virus was passaged blindly through *n* = 3 to 5 OB mice per passage. Productive infection was evident in OB mice since lung viral titers increased at each passage, with no productive infection seen in WT mice. Viral titers were quantitated via cytopathic effects, with dotted lines representing the limit of detection. (b and c) Infection of WT mice with parental H3N2 virus (p0) or obese-derived H3N2 (OBp5). (b) OB-derived H3N2 virus induced weight loss in WT mice compared to that in parental virus. Dotted line represents baseline body weight. (c) Viral titers recovered from WT mice inoculated with OB-derived H3N2 virus and assayed via hemagglutination of turkey red blood cells (*P < *0.0001, *t *= 19). Data were analyzed for panel a with ordinary one-way ANOVA, for panel b with ordinary two-way ANOVA with Sidak’s correction, and for panel c with two-tailed unpaired *t* test. Data are displayed as means ± SEM (*, *P* < 0.05). Download FIG S3, JPG file, 0.4 MB. Copyright © 2020 Honce et al. 2020 Honce et al. This content is distributed under the terms of the Creative Commons Attribution 4.0 International license.

### Obese host-passaged viruses have increased replication kinetics *in vitro*.

**FIG 3:**
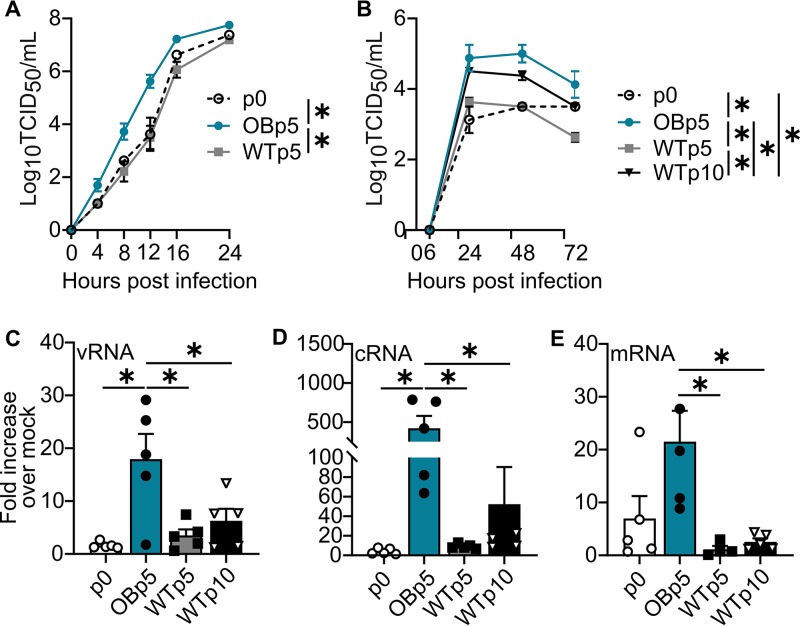
Increased replication is characteristic of obese host-passaged viruses. (A) OBp5 virus replicates more quickly in MDCK cells (*n* = 4 wells/virus) inoculated at a multiplicity of infection (MOI) of 1.0 for p0 and WTp5 viruses. (B) MDCK cells inoculated with the indicated viruses at an MOI of 0.01 (*n* = 3 wells/virus/time point). OBp5 virus replicates to higher titers than the parental p0, WTp5, and WTp10 viruses. (C to E) Mean fold increase over mock levels in RNA species production at hour 4 p.i. in MDCK cells (*n* = 5 wells/virus) inoculated with p0, OBp5, WTp5, or WTp10 virus. Total RNA was isolated, and the levels of vRNA, mRNA, and cRNA were determined in triplicate using NP strand-specific primers. OBp5 virus produces greater increases in vRNA species (C), cRNA species (D), and mRNA species than WTp5 and WTp10 viruses (E) and than p0 viruses for vRNA and cRNA species. One outlier was removed from WTp5 data for mRNA using the ROUT test for outliers. Data were analyzed for panels A and B with ordinary two-way ANOVA with Tukey’s multiple-comparison test and for panels C to E with ordinary one-way ANOVA with Tukey’s multiple-comparison test and are represented as means ± standard errors (*, *P < *0.05).

We further investigated the replicative capacity of OB and WT mouse-passaged viruses *in vitro*. Reflecting what was observed *in vivo*, OBp5 virus replicated to significantly higher titers than WTp5 (*P < *0.0001) and p0 (*P = *0.0011) viruses in single-cycle replication kinetics in MDCK cells at a multiplicity of infection (MOI) of 1.0 (Fig. 3A). Similarly, in multicycle replication kinetics (MOI of 0.001), OBp5 virus replicated to significantly increased titers versus WTp5 (*P < *0.0001), WTp10 (*P = *0.02), and p0 (*P < *0.0001) viruses (Fig. 3B). Not surprisingly, the OBp5 virus had higher vRNA (*P = *0.008), cRNA (*P = *0.01), and mRNA (*P = *0.01) levels (Fig. 3C to E), explaining its enhanced replication. DIOp5 virus showed replication kinetics similar to those of OBp5 (Fig. 2G and H). At a high MOI, DIOp5 virus replicated to significantly higher titers than p0 (*P = *0.004) and LNp5 viruses (*P < *0.0001) in MDCK cells at a high MOI. At low MOI, DIOp5 virus had higher viral titers than the p0 virus (*P = *0.01). Overall, our studies suggest that influenza infection in obese mice results in viruses with increased replicative ability. These studies show that obese host-passaged H1N1 virus had increased replication kinetics over lean host-passaged virus, supporting our hypothesis that an obesogenic environment permits evolution of increased virulence in the IAV quasispecies.

### Next-generation sequencing reveals obese host-passaged viruses have higher within-host diversity and mutations associated with virulence.

**FIG 4:**
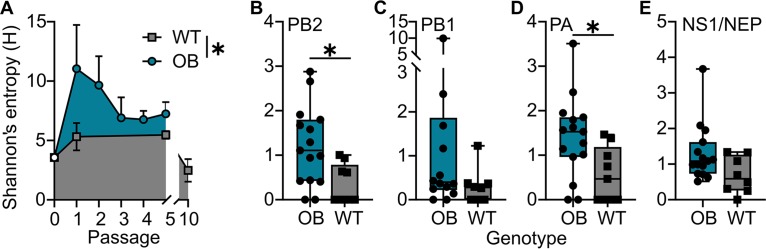
Obese host-derived viruses show increased genetic diversity. (A) OB host-derived viruses over each passage exhibit higher pooled Shannon’s entropy measurements for all genome segments than WT-derived viruses. (B to E) Mean within-host viral diversity estimated using Shannon’s entropy (*H*) for PB2 (*P = *0.0137) (B), PB1 (*P = *0.1627) (C), PA (*P = *0.0300) (D), and NEP/NS1 (*P = *0.0556) (E) gene segments. Data were analyzed for panel A with an unpaired *t* test and for panels B to E for normality using Shapiro-Wilk test, and statistical comparisons were made between using a Mann-Whitney (B, C, and E) or unpaired *t* (D) test.

**FIG 5:**
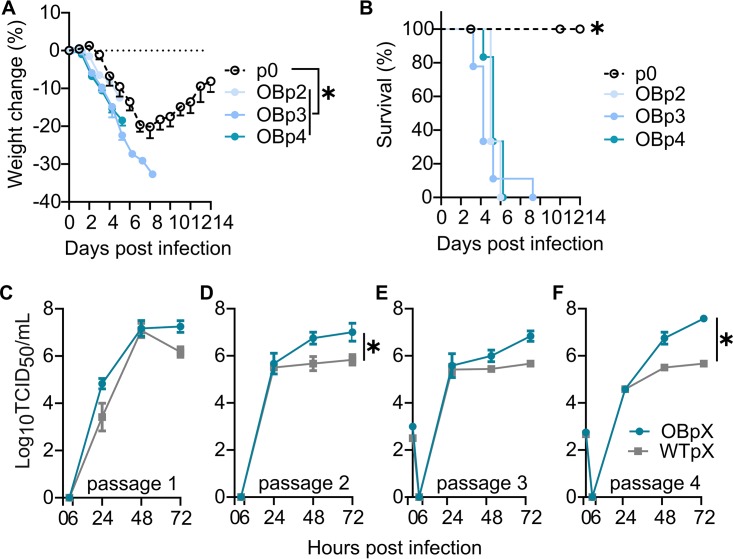
Increased virulence and replication emerge early during obese passaging. (A and B) Intermediate passages in obese mice during the experimental evolution scheme were used to inoculate WT mice. (A) Percent weight changes in WT mice inoculated with the indicated viruses. (B) Percent survival. (C to F) Early-passage OB viruses show increased replication, with OBp1 trending higher (C), OBp2 significantly replicating higher (D), OBp3 trending higher (E), and OBp4 replicating significantly higher (F) than concordant WT passage viruses. Data were analyzed for panels A and C to F with ordinary one-two ANOVA with Tukey’s multiple-comparison test and for panel B with Mantel-Cox test and are represented as means ± standard errors (*, *P < *0.05).

Obese host-passaged viruses induced greater mortality in a WT host, suggesting a viral, and not host, determinant of virulence. We used deep sequencing to analyze the viral populations of p0, OBp1 through p5, WTp1, WTp5, WTp10, DIOp1, DIOp5, LNp1, and LNp5 viruses derived from individual mouse lung homogenates. Analysis of mutations relative to p0 virus found at greater than 5% in the population showed mean within-host diversity was significantly increased in each passage in the obesity-derived populations compared to that of the lean one (Fig. 4A). The bulk of the viral diversity is explained by higher sequence variation in 2 of the 3 polymerase segments for OB-derived viruses (PB2, *P = *0.01; PB1, *P = *0.16; PA, *P = *0.03) (Fig. 5B to D). Shannon’s entropy was also higher, albeit nonstatistically significant, for NEP/NS1 (*P = *0.05) (Fig. 5E). No significant differences were found in the other four genomic segments.

Investigation of single-nucleotide variants (SNVs) revealed amino acid changes primarily in PB2, PB1, and PA proteins as well as in the NS1 protein, specifically, PB2 L154I, PB2 K482R, PA E349K, PA-X F35L, NA G336D, NS1 A202V, and NS1 R211K (Table 1). The PA-X F35L and PB2 K482R mutations are associated with mouse adaptation (39–41). No experimental studies to date have addressed the virulence properties attributable to PB2 L154I and NA G336D mutations, which were found at relatively high frequencies in OB mouse-passaged viruses, or the NS1 A202V mutation, which was found in OB mouse-passaged viruses and at a much lower frequency in WT mouse-passaged viruses. All identified variants were previously identified in circulating human H1N1 viruses, albeit at low frequencies, as evidenced by sequences in the Influenza Research Database (Table S1) (42). Finally, deep sequencing of viruses isolated from WT mice infected with OBp5 virus showed that PB2 L154I, PB2 K482R, PA E349K, and NS1 A202V variants, as well as the PA-X F35L variant associated with mouse adaptation, were maintained in the population. This contrasts with viruses from mice infected with WTp5 and WTp10 viruses, where only PB2 K482R, NS1 A202V, and PA-X F35L (WTp5) and PA-X F35L (WTp10) variants were maintained in the population (Table S2). All mutations found at 5% or greater in OB mouse-passaged viruses are displayed in Table S3.

**TABLE 1:** Minor viral variants in OB- and WT-derived viruses

Passage	SNV call^*a*^	No. (%) of mice with:																
		PB2 L154I			PB2 K482R		PA E349K		PA-X/PA F35L^*b*^		PA-X/PA F35L^*c*^		NA G33GD		NS1 A202V		NS1 R211K	
		No.^*d*^	%^*e*^		No.	%	No.	%	No.	%	No.	%	No.	%	No.	%	No.	%
p0	23.5 ± 12.5																	
OBp1	43.0 ± 14.5								2	68	2	28						
OBp2	112.3 ± 15.8								1	5					2	14	1	38
OBp3	111.3 ± 26.7	1		23					3	14	3	21			2	14	2	8
OBp4	133.7 ± 13.5	2		16	2	6			3	22	3	23	3	12	3	28	1	5
OBp5	90.8 ± 19.3	3		19	3	52	3	7	5	21	4	11	3	18	3	21	2	6
WTp1	36.4 ± 12.3				2	13					1	25						
WTp5	78.3 ± 21.8				3	50			5	38	2	35			2	8		
WTp10	85.3 ± 4.7								1	6					2	8		

a Average ± SEM number of nonsynonymous and synonymous SNVs for each passage (*n* = 3 to 8 lung homogenates/passage).

b Nucleotide change of TTT→CTT.

c Nucleotide change of TTT→TTG.

d Total number of mice with ≥5% variants.

e Average relative frequency of mutation in samples with mutation.

10.1128/mBio.03341-19.4 TABLE S1 Relative frequencies of obese host-derived mutations found in circulating human H1N1 viruses. Download Table S1, DOCX file, 0.01 MB. Copyright © 2020 Honce et al. 2020 Honce et al. This content is distributed under the terms of the Creative Commons Attribution 4.0 International license.

10.1128/mBio.03341-19.5 TABLE S2 Previously identified viral variants present upon reinfection of WT mice with indicated viruses. Download Table S2, DOCX file, 0.01 MB. Copyright © 2020 Honce et al. 2020 Honce et al. This content is distributed under the terms of the Creative Commons Attribution 4.0 International license.

10.1128/mBio.03341-19.6 TABLE S3 Amino acid variants in OB- and WT-passaged viruses found at 5% relative frequency or more. Download Table S3, DOCX file, 0.05 MB. Copyright © 2020 Honce et al. 2020 Honce et al. This content is distributed under the terms of the Creative Commons Attribution 4.0 International license.

Within-host diversity was less variable in the DIO versus LN model. However, all DIO-derived viruses, as with OB-derived viruses, exhibited greater Shannon’s entropy than those isolated from LN hosts (*P = *0.04) (Fig. S2I). For DIO passaging, the single SNV biologically relevant in DIOp5 was PB2 E158G, which has been previously reported in human samples (Table S1). All variants found at 5% or greater are in Table S4.

10.1128/mBio.03341-19.7 TABLE S4 Amino acid variation in DIO- and LN-passaged viruses found at 5% relative frequency or more. Download Table S4, DOCX file, 0.04 MB. Copyright © 2020 Honce et al. 2020 Honce et al. This content is distributed under the terms of the Creative Commons Attribution 4.0 International license.

### Increased pathogenicity occurs at early passages through the obese model.

Early-passage viruses contained mutations also present in the OBp5 virus, with NS1 R211K and NS1 A202V mutations present beginning at OBp2; PB2 L154I mutation arising at OBp3; and PB2 K482R and NA G336D mutations arising at OBp4. Only the PA E349K variant was unique to OBp5 (Table 1). Thus, we questioned how early the increased virulence phenotype arises in the OB model. WT mice were inoculated with 80 TCID_50_ of OBp2, OBp3, and OBp4 viruses, which is close to the MLD_50_ of the OBp5 virus (10^1.92^ TCID_50_). All viruses induced significant weight loss (Fig. 5A) and mortality (Fig. 5B) in WT mice. As a comparison, OBp5 virus was 100% lethal by day 8 p.i. in WT mice at 300 TCID_50_. The early-passage viruses induced 100% lethality by day 10 p.i. at a dose similar to that of the OBp5 virus MLD_50_, showing that the increase in mortality in WT mice occurs early after viral passaging through the obese mouse (Fig. 2C and 5B).

Increased replication was also observed with early-passage viruses. Multistep replication kinetics (MOI of 0.001) in MDCK cells showed replication trended higher in OBp1 than WTp1 (*P = *0.06) (Fig. 5C), was significantly increased for OBp2 versus WTp2 (*P = *0.02) (Fig. 5D), trended higher for OBp3 versus WTp3 (*P = *0.05) (Fig. 5E), and was significantly increased for OBp4 versus WTp4 (*P = *0.0007) (Fig. 5F).

### Robust IFN responses restrict viral diversity and acquisition of virulent viral phenotypes.

**FIG 6:**
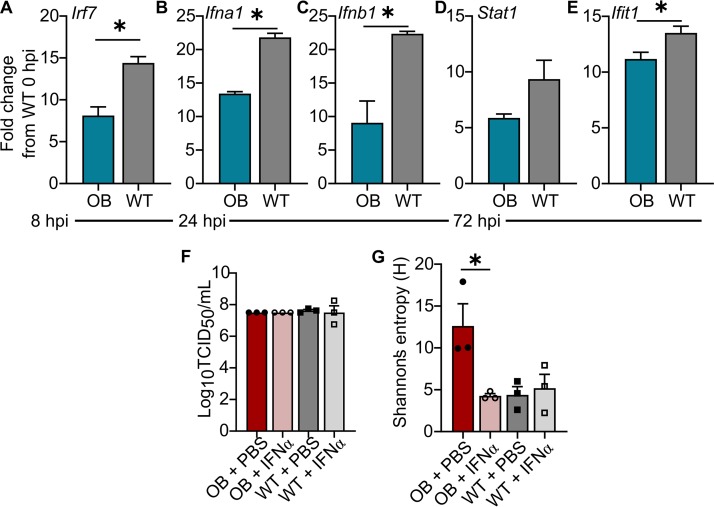
Robust interferon responses restrict viral diversity and acquisition of virulent viral phenotypes. (A to E) OB and LN mice (*n* = 3/time point) were intranasally inoculated with 10^3^ TCID_50_ of CA/09 virus. RNA was extracted and HT-qPCR was performed on cDNA using the Fluidigm platform, with expression normalized to that of β-actin and average expression of the target gene in WT mice at baseline. Compared to WT mice, OB mice had reduced expression of *Irf7* at hour 8 p.i. (A), *Ifna1* (B) and *Ifnb1* (C) at hour 24 p.i., and *Stat1* (D) and *Ifit1* (E) at hour 72 p.i. (F and G) OB and WT mice were treated with recombinant Ifna2 protein and inoculated with 10^3^ TCID_50_ of CA/09 virus. (F) Ifna2 treatment did not reduce viral titers at day 3 p.i. (G) Ifna2 treatment reduced viral population diversity in OB-derived viruses at day 3 p.i. Data shown in panels A to E were analyzed with unpaired *t* test and in panels F and G with ordinary one-way ANOVA with Tukey’s correction. Data are represented as means ± standard errors (*, *P < *0.05).

Obese hosts have impaired cellular immune responses that may translate to reduced control of viral spread and clearance of viral infection (7). Our studies demonstrated increased viral diversity occurred in obese mice by day 3 p.i. At this early time point, interferons (IFN) are critical for controlling viral replication and spread (32). Thus, we hypothesized that the emergence of a diverse, virulent IAV quasispecies in OB mice was due to a blunted IFN response. To test this, total RNA was extracted from lungs of WT and OB mice inoculated with 10^3^ TCID_50_ of CA/09 virus and probed for type I IFN signaling. Analysis of quantitative PCR (qPCR) showed IFN induction and signaling genes were blunted in the obese host. At 8 h p.i., there is significantly less *Irf7* (*P = *0.003) (Fig. 6A), leading to reduced expression of *Ifna1* (*P = *0.0001) (Fig. 6B) and *Ifnb1* (*P = *0.007) (Fig. 6C) at hour 24 p.i. By 72 h p.i., this translated to reduced *Stat1* (*P = *0.09) (Fig. 6D) and significantly reduced *Ifit1* (*P = *0.03) (Fig. 6E) expression in the lungs of OB mice compared to WT mice. All gene expression was standardized to levels of WT mice at baseline.

We then investigated whether the emergence of a diverse, virulent IAV quasispecies is sensitive to IFN. WT and OB mice were primed via oral gavage with 1,000 U recombinant murine IFN-α2 protein, 8 h later were infected with CA/09 virus, and then were intranasally treated with 1,000 U IFN-α2 at 8 h p.i. While there was no reduction in lung viral titers (Fig. 6F), there was a significant decrease in Shannon’s entropy of single-nucleotide polymorphism variants in infected OB mice versus mice mock treated with PBS at day 3 p.i. (Fig. 6G). No difference in Shannon’s entropy was found in WT-treated mice. The lack of modulation in viral titers due to IFN-α2 treatment most likely is due to the early stage of infection monitored, since viral titers do not differ in untreated WT and OB mice at this early time point as well (Fig. 1A and 6F). We speculate that the IFN-α2 treatment impacts viral titers, spread, and overall survival at later time points. Our studies demonstrate that the blunted IFN response associated with obesity support the emergence of viral variants that could impact virulence.

### Human primary respiratory epithelial cells from obese donors show increased influenza virus replication and blunted interferon responses.

**FIG 7:**
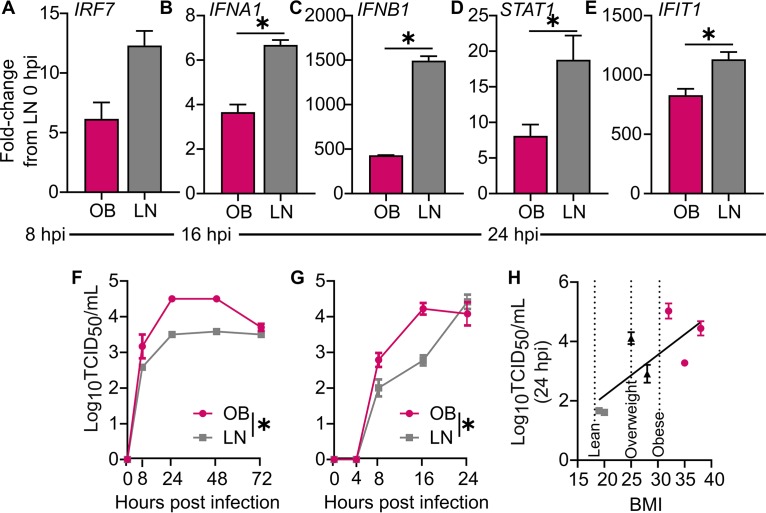
Human primary respiratory epithelial cells from obese donors show increased influenza virus replication and blunted interferon responses. (A to E) RNA extracted from obese and lean host-derived NHBE cells (*n* = 3 wells/time point) infected at an MOI of 10 with CA/09 virus. Shown are gene expression normalized to that of *GAPDH* and average expression of target genes in lean cells at baseline. Compared to lean host-derived cells, obese host-derived NHBE cells had reduced expression of *IRF7* at hour 8 p.i. (A), *IFNA1* (B) and *IFNB1* (C) at hour 16 p.i., and *STAT1* (D) and *IFIT1* (E) at hour 24 p.i. (F to H) Obese host-derived NHBE cells (*n* = 12 wells/donor) inoculated with CA/09 virus showed increased replication kinetics compared to those of lean host-derived cells. (F and G) Representative age-, race-, and sex-matched, BMI-discordant NHBE cells infected at an MOI of 0.01 with CA/09 virus (F) and MOI of 10 with CA/09 virus (G). Lean cells were derived from a 52-year-old white male with a BMI of 25, and obese cells derived from a 53-year-old white male with a BMI of 38. (H) Viral titers at hour 24 p.i. from a panel of BMI-discordant NHBE cells (*n* = 3 wells/donor; *R*^2^=0.4708). Data were analyzed for panels A to E with unpaired *t* test, for panels F and G with ordinary one-way ANOVA, and for panel H with linear regression. Data are represented as means ± standard errors (*, *P < *0.05).

We then assayed whether the delayed IFN response observed in obese mice (both genetic and DIO) translated to humans. Diminished IFN responses were found in differentiated normal human bronchial epithelial (NHBE) cells derived from obese donors. Infecting body mass index (BMI)-discordant NHBE (i.e., obtained from people of the same sex, race, and age but different BMI) cells at the air-liquid interface with CA/09 virus at an MOI of 5 resulted in reduced expression of *IRF7 (P = *0.08) (Fig. 7A) at hour 8 p.i., significantly blunted *IFNA1* (*P = *0.002) (Fig. 7B) and *IFNB1* (*P = *0.0001) (Fig. 7C) at hour 16 p.i., and reduced IFN signaling, as *STAT1* (*P=* 0.03) (Fig. 7D) and *IFIT1* (*P = *0.02) (Fig. 7E) expression at hour 24 p.i. was reduced in obese host-derived versus lean host-derived NHBE cells. All gene expression was normalized to levels in lean host-derived cells at baseline. These impaired IFN responses were accompanied by increased viral replication. CA/09 virus replicated to higher titers in obese host-derived NHBEs at both low (*P < *0.0001) (Fig. 7F) and high (*P = *0.003) MOI (Fig. 7G) compared to replication in lean host-derived, age-, race-, and sex-matched NHBE cells. Indeed, viral titers positively correlated with BMI (*P = *0.05) (Fig. 7H and Table S5). These studies provide initial evidence that the epithelial cells from obese people have blunted antiviral responses, leading to increased viral replication kinetics. Further studies are needed to assess how this impacts viral diversity.

10.1128/mBio.03341-19.8 TABLE S5 NHBE cells used in study. Download Table S5, DOCX file, 0.02 MB. Copyright © 2020 Honce et al. 2020 Honce et al. This content is distributed under the terms of the Creative Commons Attribution 4.0 International license.

## DISCUSSION

A growing body of literature suggests that obesity impairs the antiviral response to influenza A virus and other viral, bacterial, fungal, and parasitic infections (3, 43). However, little is known of the impact an obese host has on the virus itself. In this study, we used experimental passaging of an H1N1 virus through two models of obesity, both DIO and OB, and observed increased virulence upon infection of a WT host compared to infection by WT and lean mouse-passaged viruses. Further, we demonstrated that OB mice were productively infected with a human seasonal H3N2 virus (A/Switzerland/9715293/2013), and the resulting passage 5 virus caused disease in a WT host. This change in viral phenotype was also observed with viruses derived from infection of obese host-derived NHBEs. Taken together, these data provide strong evidence that viral infection in an obesogenic environment characterized by reduced interferon pressures results in rapid changes to the viral population, some of which lead to increased virulence in mice.

Our findings complement evidence showing within-host evolution of viral pathogens is sensitive to host characteristics. The obesogenic environment mirrors other immunocompromised states, including undernourished, aged, and immunosuppressed hosts (44, 45). Host nutritional status is a vital component of the body’s response to pathogens, as is seen in hospitalized adults with influenza-like illness, in which both underweight and overweight status impacts susceptibility to IAV (23). In work with malnourished mice, Beck et al. described heightened host oxidative stress as an influence on mutation rates of RNA viruses, and it is known RNA sustains oxidative damage that may directly impact RdRp fidelity (29, 46, 47). The obesogenic environment generates high levels of reactive oxygen species (ROS), which may contribute to the distinct evolutionary pressures in the obese state (48, 49). Obesity has been proposed to trigger premature aging of the immune system (45, 50, 51), mirroring an immunocompromised state, and both aged (30) and immunocompromised (52, 53) hosts promote the emergence of novel viral variants. With blunted immune responses (7, 54) and extended viral shedding (20, 22) in obese humans, the same phenomenon may occur with IAV. Our evidence, and complementary evidence previously reported in the literature (15, 28), shows that a delayed interferon response early in infection permits increased viral replication and spread (17, 55) and may lead to the increased viral diversity and emergence of virulent variants observed in both obese mice and obese host-derived primary human respiratory cells.

Influenza virus infections are notably heterogenous, and many of the minor variants in obese donor-passaged viruses have been reported to increase virulence in mice (56). While no variant reached consensus in the obese host-passaged viruses, among the viral segments there was an increase in mean genetic diversity. Across biological replicates and passaging in two models of obesity, we saw similar phenotypes of increased replication and genotypic variants arising in polymerase segments and NS1. Minor variants emerged in the OBp5 virus that have been associated with increases in replication, specifically the variants PA-X F35L (39, 40), PB2 K482R (41), and PB2 E158G (57–59). Changes to the E349 site in PA also result in increased polymerase activity (57, 60, 61). We observed trends toward higher genetic diversity in NS1, a protein at the forefront of viral life cycle regulation and antagonism of the host antiviral response (62). The NS1 R211K mutation also emerged in IAV-infected allogenically pregnant mice, a model for infection during human gestation (63). This change resulted in increased polymerase activity and reduced cytokine and interferon production, with recombinant viruses causing increased mortality and morbidity in WT, nonpregnant mice, mirroring what was seen here with our obese host-derived viruses (63). While we observed fewer minor variants in the DIO model, a similar phenotype emerged, with most variants occurring in the polymerase and NS1 segments. The DIOp5 change of PB2 E158G, also found in OBp1, increases viral replication and infection severity *in vivo* and *in vitro* (58, 64). We found no previous experimental studies identifying NA G336D and NS1 A202V variants as possible mediators of virulence. We propose the OB environment affords a relaxed bottleneck, thereby increasing the scope of viral variants contributing to virulence. The lack of early immune pressures highlighted by reduced type I interferon gene expression in the OB host may allow certain viral variants to thrive that would otherwise be limited due to the robust WT immune response, with select variants stable after infection of WT mice (Table S3). This altered intrahost quasispecies may contribute to the increased viral spread and disease severity observed in the obesogenic state (7, 14, 18).

With a greater scope of potential minor variants, the OB host may allow a variety of minor amino acid changes to emerge, impacting viral replication and disease severity. Studies with poliovirus showed that a greater scope of viral variants increases *in vivo* virulence, with high-fidelity RdRp mutants showing little pathogenesis (65, 66). However, poliovirus mutators that have 2- to 3-fold higher rates of mutations are driven to extinction by WT virus (67). These results point to a balance between mutations improving within-host fitness and the detrimental impacts of low polymerase fidelity. Further, another model, proposed by Fitzsimmons et al., states that increased replicative speed is under selective pressure; thus, high mutation rates are favored (68). In the current study, we have shown that OB-derived viruses grow to higher titers at early time points in both single- and multicycle replication, possibly by exploiting the reduced induction of the interferon response (7). By later time points, there was no significant difference in viral titers among p0 or WT- or OB-passaged viruses. The OB environment may generate a more robust viral population early during infection, providing a key fitness advantage to increase viral spread before recognition by the host immune response (55). Once the delayed immune response is initiated, the variants selected for in the OB environment may provide no further advantage. However, this early advantage may prove biologically significant, as evidenced by decreased survival in OBp5-inoculated WT mice. Further studies should investigate properties of the obese lung microenvironment and physiology that promote viral growth and impact disease severity, as well as the observed increases in viral spread with no concordant significant increase in viral titers.

Overall, our results demonstrate the use of the obese mouse model as a tool for experimental adaptation of IAV, and it raises questions concerning the natural within-host evolution of the IAV quasispecies in the increasingly obese population. Our studies indicate infection of an obese host resulted in minor variant changes that could impact pathogenicity. The virulence properties appeared early in mouse passaging, with amino acid-level changes occurring after a single passage in the obesity environment. Our findings translated to *ex vivo* human samples. Consistent with our mouse studies, we demonstrated that CA/09 virus replicated with faster kinetics and to higher titers in primary NHBE cells obtained from people with higher BMI.

With increasing demands for efficacious IAV antivirals and vaccines, understanding how the obese host will respond is important; however, even more important may be discerning the impact the obese population will have on potentially increasing the virulence of the IAV quasispecies (69, 70). Our studies have suggested that a reduced interferon response is partially responsible for the lack of early viral control, permitting the emergence of virulent IAV strains, since we report differential levels of interferon and ISG expression in OB mice up to collection of passaged virus at day 3 p.i. Ongoing studies must continue investigating unique properties of the obesogenic microenvironment that permit the emergence of a more virulent population, because this has implications for antigenic drift and the emergence of antiviral-resistant and vaccine escape variants in the obese population (71).

## MATERIALS AND METHODS

### Animal studies.

Eight- to 10-week-old WT male C57BL/6 (JAX 000664) and B6.C-*Lep^ob/^*^ob^/J genetically obese (OB) (JAX 000632) mice were obtained from The Jackson Laboratory. Male B6 mice were used, as they are more susceptible to diet-induced obesity than female mice (72). All animals were provided with food and water *ad libitum.* For the diet-induced obesity model, 4-week-old male, C57BL/6 mice were randomly assigned to either a high-fat (60% kcal%; no. D12492) or low-fat (10% kcal%; no. D12450B) diet (Research Diets) fed *ad libitum* for 12 to 14 weeks to generate diet-induced obese (DIO) or lean (LN) mice, respectively. Water was provided *ad libitum*. All procedures involving animals were approved by the St. Jude Children’s Research Hospital Institutional Animal Care and Use Committee and followed the *Guide for the Care and Use of Laboratory Animals* (73).

### Viral strains.

A/California/04/2009 (H1N1) and A/Switzerland/9715293/2013 (H3N2) parental viruses were obtained from Robert Webster (St. Jude) and propagated in the allantoic cavity of 10-day-old specific-pathogen-free embryonated chicken eggs as described previously (74). Parental viruses considered p0 include pH1N1 at egg passage 2 and H3N2 at egg passage 3. A/California/04/2009-PA nLuc (CA/09-nLuc) was rescued by using the 8-plasmid 293T/MDCK coculture system as previously described (33, 34). All work was completed at biosafety level 2+ and animal biosafety level 2. These studies were halted from fall 2011 until December 2014 during the gain-of-function research moratorium.

### Cell lines and primary culture.

Madin-Darby canine kidney cells (MDCK cells; RRID:CVCL_0422) were maintained in Dulbecco’s minimum essential medium (DMEM; Lonza) supplemented with 2 mM GlutaMAX (Gibco) and 10% fetal bovine serum (FBS; Atlanta Biologicals) and grown at 37°C under 5% CO_2_. For *in vitro* inoculations with the indicated viruses, serum-free DMEM was supplemented with 0.075% bovine serum albumin (BSA; Gibco) and 1 μg/ml tosylsulfonyl phenylalanyl chloromethyl ketone (TPCK)-treated trypsin. Normal human bronchial epithelial cells (number CC-2540S; NHBE cells) were obtained commercially (Lonza) and cultured in bronchial epithelial cell growth basal medium (BEBM) supplemented with bronchial epithelial cell growth medium (BEGM) SingleQuots. NHBE cells were seeded onto permeable transwell inserts at a concentration of 2.5 × 10^4^ cells per well. After reaching confluence, apical medium was aspirated, and medium in the basolateral chamber was replaced with air-liquid interface medium containing half DMEM and half BEBM, plus the BEGM SingleQuots to complete differentiation. Medium was changed every 2 to 3 days.

### Determination of viral titers.

Viral titers were determined by 50% tissue culture infectious dose (TCID_50_) assays in MDCK cells as previously described (74) and scored by hemagglutination (HA) endpoint or the appearance of cytopathic effects (CPE) at day 3 p.i. (75). Infectious viral titers were calculated using the Reed-Muench method (76).

### Bioluminescent imaging.

Mice (*n* = 8 per group) were deeply anesthetized with isoflurane and retroorbitally injected with 0.3 μl/g Nano-Glo substrate (Promega) diluted in 100 μl of PBS and immediately sacrificed. Whole lungs were removed and imaged for 3 min using a Xenogen IVIS200 system with Living Image software (PerkinElmer). Living Image software was used to quantify viral spread. Data are representative of three independent experiments.

### Experimental evolution of IAV.

Lean (WT or LN) and obese (DIO or OB) mice (*n* = 3 to 5/group) were lightly anesthetized under 2.5% isoflurane and intranasally inoculated with p0-H1N1 at 10^3^ TCID_50_ in 25 μl of phosphate-buffered saline (PBS) as described previously (76). Mice were observed daily or twice daily during the height of infection for morbidity and weight loss. Morbidity was scored by at least 2 people using the following clinical score measurements: 0, normal; 1, ruffled fur; 2, hunched back, slow circular movement; 3, trembling; 4, paralysis and moribund. At day 3 p.i., mice were sacrificed under CO_2_ asphyxiation and lungs were collected and homogenized in PBS. Viral titers were determined as described above, and pooled lung homogenate standardized to a viral dose of 10^3^ TCID_50_/ml in PBS was used to infect a new set of lean or obese mice for a total of 5 or 10 passages (Fig. 1F). We did not attempt to amplify or sucrose purify viruses from the lung homogenate of each passage. Further, matrix effects of the obese and lean lung homogenate impacting our findings are minimal, as less than 20% of lung homogenate was used as part of the inoculum at each passage. However, we cannot discount that host factors in the lung homogenate could impact subsequent observations and is a caveat to this study. H3N2 passaging was accomplished as described above, except H3N2-p0 virus was used without dilution to inoculate 3 OB and WT male mice. At day 3 p.i., lungs were collected and homogenized. Undiluted lung homogenate (25 μl) was used for the next round of infection. A total of 5 passages were performed. A second biological replicate of this study was performed, and all data are representative of 2 independent experiments.

### Reinfection experiments.

Quantification of the virulence of WT, LN, OB, and DIO mouse-passaged viruses was accomplished by intranasally infecting *n* = 5/virus/dilution 10-week-old C57BL/6 with p5 viruses at log_10_ dilutions from 10^2^ to 10^5^ TCID_50_ in 25 μl of PBS to determine the 50% mean lethal dose (MLD_50_). Mice were euthanized when they reached the humane endpoint of greater than 30% weight loss and clinical scores of 4. To understand the disease progression of the passaged viruses, 5 recipient WT mice were infected intranasally with 300 TCID_50_/ml p0, OBp5, WTp5, and WTp10 viruses. Mice were monitored for morbidity over 14 days. On day 3 p.i., lungs were collected from 3 mice and homogenized for viral titer determination as described above.

### *In vitro* replication kinetics.

Infections were performed as described previously (74). Briefly, MDCK cells were infected at the indicated multiplicity of infection (MOI) for 1 h. Cells were washed three times with PBS to remove unbound virus, and infected cells were cultured in DMEM containing BSA and TPCK-trypsin. Aliquots of culture supernatants were collected at noted time points and immediately stored at –80°C for the determination of virus titers as described above.

### Fluidigm HT-qPCR.

Total RNA was extracted from whole-lung homogenate using a QIAshredder (number 79654; Qiagen) and RNeasy minikit (number 74104; Qiagen) with DNase treatment. cDNA was synthesized using 500 ng total RNA using SuperScript VILO (number 11754050; Invitrogen). cDNA (1 μl) and primers were loaded onto a 96- by 96-well chip for high-throughput qPCR (HT-qPCR) on the Fluidigm platform, with downstream analysis completed in Fluidigm real-time PCR analysis software and Microsoft Excel. Resulting threshold cycle (*C_T_*) values were normalized to endogenous *GAPDH* expression and Δ*C_T_* values to average expression of target genes in lungs of WT mice at baseline.

### RNA extraction and qPCR.

BMI-discordant NHBEs were inoculated with p0-CA/09 virus at an MOI of 10, with RNA extracted at the indicated time points using TRIzol (Ambion) according to the manufacturer's specifications. RNA was stored at –80°C prior to the generation of cDNA using SuperScript VILO and qPCR using the QuantiFast SYBR green kit (number 204054; Qiagen) and primer assays for *IFNA1* (number QT00201964), *IFNB1* (number QT00203763), and *IFIT1* (number QT00201012). Resulting *C_T_* values were normalized to endogenous *GAPDH* expression (number QT00079247) and Δ*C_T_* values to average expression of target genes in LN NHBEs at hour 0 p.i., with ΔΔ*C_T_* values reported as fold changes over baseline.

### v/c/mRNA analysis.

Total RNA was extracted from MDCK cells inoculated with p0, pooled OBp5, WTp5, and WTp10 viruses at an MOI of 0.001 using TRIzol (Ambion) according to the manufacturer’s specifications. Complementary cDNAs to viral RNA (vRNA), complementary RNA (cRNA), and mRNA were synthesized using primers specific to the viral NP gene as described previously (77). Real-time PCR was performed using a QuantiFast SYBR green PCR kit (number 204054; Qiagen) on a CFX96 real-time system (Bio-Rad) as described previously (74).

### Interferon treatment and infection.

Eight-week-old male OB and C57BL/6 WT mice were intranasally infected with 10^3^ TCID_50_ of p0 in 25 μl PBS. Eight hours preinfection, mice were primed with a 100-μl oral gavage of either 1,000 U recombinant murine IFN-α2 (number 14831262; ThermoFisher) or vehicle control (PBS plus 0.1% BSA). Eight hours postinfection, mice were lightly anesthetized with isoflurane and intranasally treated with 1,000 U recombinant murine IFN-α or vehicle control in 25 μl. Weights and clinical scores were monitored daily for 14 days. Lungs (*n* = 3 mice per group) were collected at day 3 p.i. and homogenized in PBS with viral titers quantified via TCID_50_. Viral diversity was calculated through deep sequencing and measurements of Shannon’s entropy.

### Deep-sequencing preparation.

Viral RNA was extracted from 50 μl of whole lung homogenate on a Kingfisher Flex magnetic particle processor (Thermo Fisher Scientific) by using the Ambion MagMAX-96 AI/ND viral RNA isolation kit (number AM1834; Applied Biosystems). RNA concentration was measured spectrophotometrically (NanoDrop). Multisegment reverse transcription-PCR (MS RT-PCR) was performed using a SuperScript III one-step RT-PCR system with Platinum *Taq* high-fidelity DNA polymerases (number 12574-035; ThermoFisher) and an influenza-specific universal set of primers (77) (Opti-F1, 5′-GTTACGCGCCAGCAAAAGCAGG-3′; Opti-F2, 5′-GTTACGCGCCAGCGAAAGCAGG-3′; Opti-R1, 5′-GTTACGCGCCAGTAGAAACAAGG-3′). RNA (5 μl) was added and placed into a thermocycler paused at 55°C. The following cycling parameters then were followed: 1 cycle of 55°C for 2 min; 1 cycle of 42°C for 60 min; 94°C for 2 min; 5 cycles of 94°C for 30 s, 44°C for 30 s, and 68°C for 3.5 min; 26 cycles of 94°C for 30 s, 57°C for 30 s, and 68°C for 3.5 min; 1 cycle of 68°C for 10 min; and then a hold at 4°C. Five microliters of the reaction mixture was analyzed by 0.8% agarose gel electrophoresis to verify all genomic segments were present, with PB1 and PB2 migrating together at 2.3 kb and minimal nonspecific amplification below 800 bp. The MS RT-PCR was purified using an Agencourt AMPure XP kit (Beckman Coulter) according to the manufacturer’s instructions. Briefly, 40 μl of the DNA amplicons was transferred to a 96-well microplate (Bio-Rad) and mixed with Agencourt AMPure XP beads (Beckman Coulter) for magnetic separation of the amplicons. The bound beads were washed twice with 100% ethanol, and the purified amplicons were suspended in 1× Tris-EDTA buffer. The concentration of the purified DNA was measured spectrophotometrically prior to storage at –20°C. DNA amplicons were deep sequenced using Illumina MiSeq technology. Deep sequencing was performed by the St. Jude Children’s Research Hospital Hartwell Center, with DNA libraries prepared using Nextera XT DNA-Seq library preparation kits (number FC-131-1024; Illumina) with 96 dual-index bar codes and sequenced on an Illumina MiSeq personal genome sequencer.

### Deep-sequencing bioinformatics and analysis.

Single-nucleotide variants (SNVs) relative to the reference sequence [A/California/04/2009 (H1N1)] were determined by two independent methods. First, reads were mapped using the breseq pipeline. breseq was run with the –predict-polymorphisms option to detect polymorphic mutations, the –no-junction-prediction option to omit junction prediction, and the –targeted-sequencing option to account for uneven coverage due to the segmented influenza genome. Mapping results were tabulated using the gdtools utility distributed with breseq (78, 79). Second, reads were mapped to the reference sequence using CLC Genomics Workbench 9 (Qiagen, Denmark) as previously reported (80, 81). Briefly, fastq files were imported, and sequences were trimmed to remove low-quality (<Q30) bases and reads shorter than 50 bases. The reads were aligned to the reference genome (accession numbers MN371615, MN371613, MN371611, MH393827, MN371617, MN596847, MN596849, and MN371614), and the low-frequency-variant detection tool was used to identify SNVs at greater than 1% relative frequency compared to the original inoculum (p0). Variants were filtered to remove low-quality variants, and results were compared with the previous bioinformatics workflow. No significant difference was detected between the two methods, increasing our confidence in variant calling. Variants reported here used the first method (breseq).

Within-host diversity was measured using Shannon’s entropy (*H*):

$$H\left(x\right)=\overset{n}{\underset{i}{\sum }}P(i)\log_{2}P(i)$$

where *P*(*i*) is the relative frequency of each single-nucleotide variant (82). Diversity calculations were performed individually across gene segments with absolute values reported.

### Querying databases.

The Influenza Research Database was used to determine whether the variants emerging within the study have been previously identified in human surveillance (42). Protein sequence variance analysis for human H1N1 was performed for segment 1 (PB2), segment 3 (PA and PA-X), segment 6 (NA), and segment 8 (NS1). Comparison of the variants discovered was made in reference to A/California/04/2009 virus, and relative variant frequencies were calculated by dividing the number of the identified amino acid minor variants by the total number of sequences queried.

### Statistical analysis.

Data were organized in Microsoft Excel and GraphPad Prism 8. Specifics of statistical details for each experiment are in the figure legends. All data are displayed as means ± standard errors of the means with in-text statistical significance designated when appropriate for multiple comparisons and in figure legends for outputs of chosen statistical tests. Outliers are indicated with an “X” and determined using the ROUT test for outliers set at *Q* = 1%. Significance for all experiments was set at α = 0.05 and is indicated by an asterisk. Bioinformatics analysis was completed using the R statistical package and CLC genomics. The cutoff for minor variants was set at 5%.

### Data availability.

Deep-sequencing data sets have been deposited in the NCBI Sequence Read Archive (SRA) under BioProject identifier PRJNA589188, with SUB6541476 containing primary infection data and SUB6637573 containing reinfection data.
