# Supplementary figures and images for: Obesity-Related Microenvironment Promotes Emergence of Virulent Influenza Virus Strains
Source: mBio. 2020 Mar 3;11(2):e03341-19. doi: 10.1128/mBio.03341-19 (PMC7064783; doi:10.1128/mBio.03341-19)

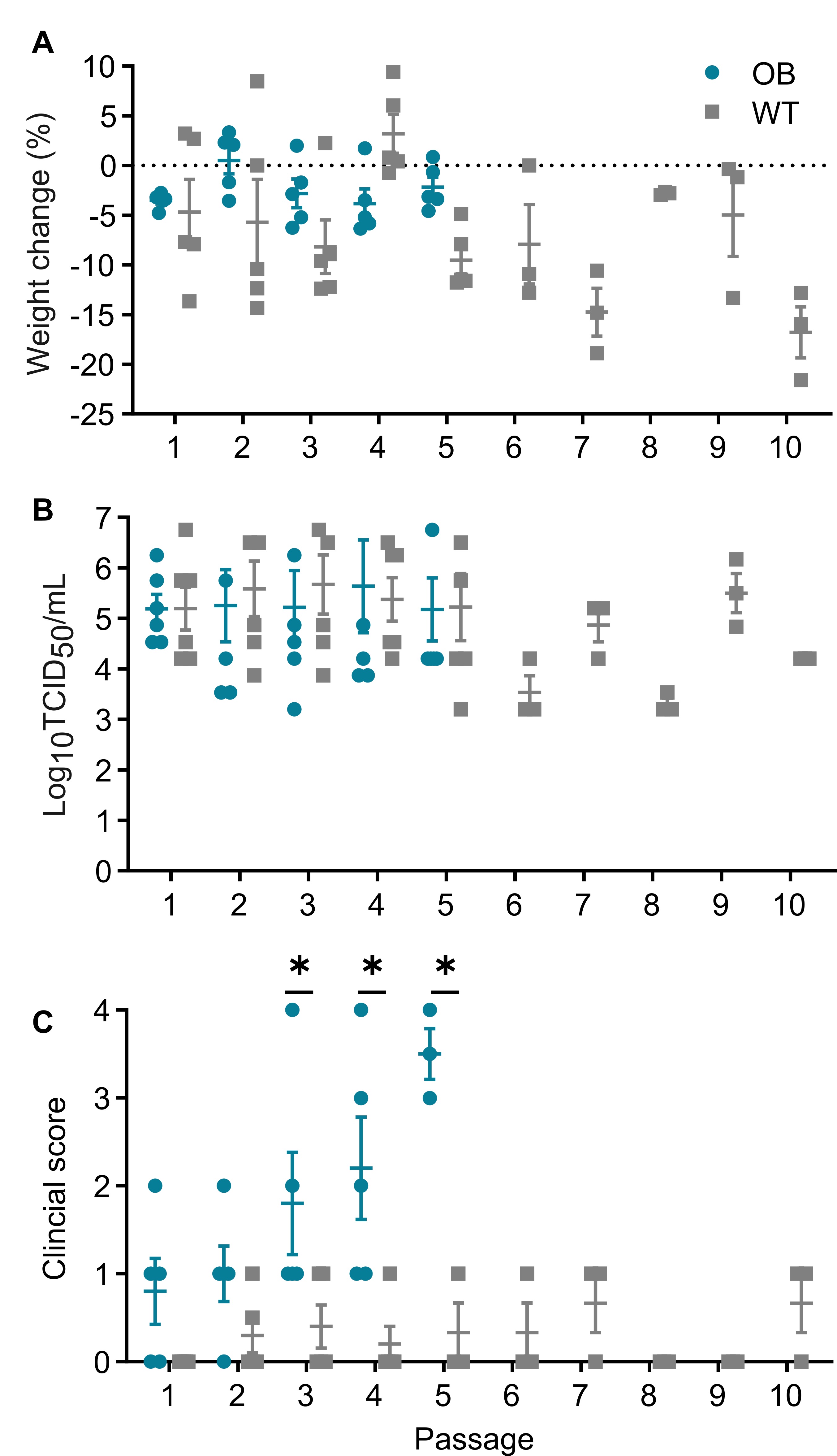

Supplement: FIG S1 [file mBio.03341-19-sf001.jpg]

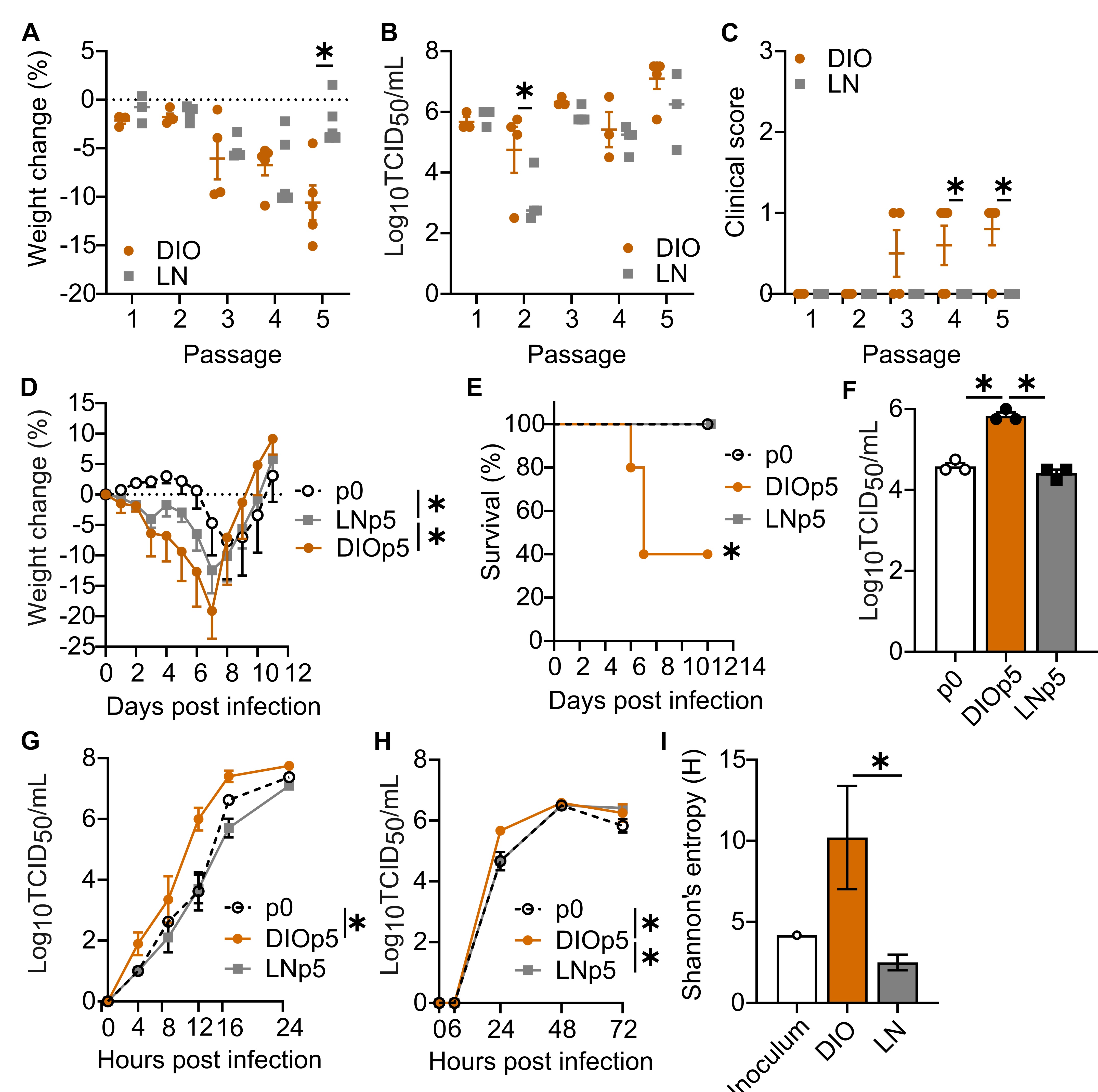

Supplement: FIG S2 [file mBio.03341-19-sf002.jpg]

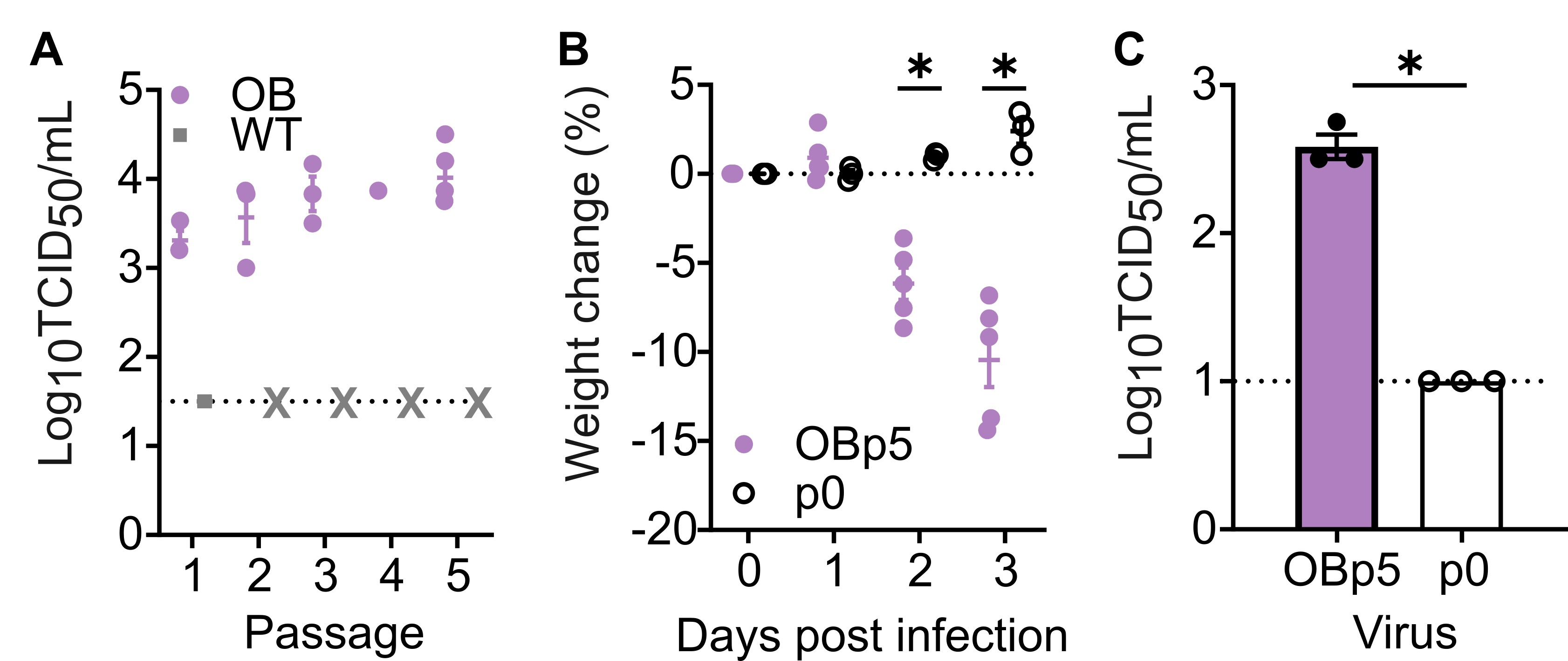

Supplement: FIG S3 [file mBio.03341-19-sf003.jpg]
