# Supplementary material for: Obesity-Related Microenvironment Promotes Emergence of Virulent Influenza Virus Strains
Source: mBio. 2020 Mar 3;11(2):e03341-19. doi: 10.1128/mBio.03341-19 (PMC7064783; doi:10.1128/mBio.03341-19)
Supplement: TABLE S1 [file mBio.03341-19-st001.docx]

Supplementary Table 1. Relative frequencies of obese-derived mutations found in circulating human H1N1 viruses.

| Protein | Amino Acid Position | Consensus Amino Acid | Variant Amino Acid | Sequences with Variant | Total Sequences | Relative Frequency of Variant (%) |
| --- | --- | --- | --- | --- | --- | --- |
| PB2 | 154 | Leu | Ile | 114 | 11321 | 1.01 |
| PB2 | 158 | Glu | Gly | 3 | 11322 | 0.03 |
| PB2 | 482 | Lys | Arg | 15 | 11347 | 0.13 |
| PA-X | 35 | Phe | Leu | 18 | 14283 | 0.12 |
| PA | 35 | Phe | Leu | 17 | 13998 | 0.12 |
| PA | 349 | Glu | Lys | 7 | 11402 | 0.06 |
| NA | 336 | Gly | Asp | 138 | 20109 | 0.69 |
| NS1 | 202 | Ala | Val | 2 | 11122 | 0.02 |
| NS1 | 211 | Arg | Lys | 21 | 11112 | 0.19 |

Influenza Research Database (Fludb) accessed 11 August 2019.
