# Supplementary material for: Obesity-Related Microenvironment Promotes Emergence of Virulent Influenza Virus Strains
Source: mBio. 2020 Mar 3;11(2):e03341-19. doi: 10.1128/mBio.03341-19 (PMC7064783; doi:10.1128/mBio.03341-19)
Supplement: TABLE S2 [file mBio.03341-19-st002.docx]

**Supplementary Table 2. Previously identified viral variants present upon reinfection of WT mice with indicated viruses.**

| Passage | PB2  L154I | | PB2  K482R | | PA  E349K | | PA-X F35L^a^ | | PA-X F35L^b^ | | NA  G336D | | NS1  A202V | | NS1 R211K | |
| --- | --- | --- | --- | --- | --- | --- | --- | --- | --- | --- | --- | --- | --- | --- | --- | --- |
|  | #^c^ | %^d^ | # | % | # | % | # | % | # | % | # | % | # | % | # | % |
| OBp5 | 2 | 45% | 5 | 55% | 5 | 8% | 7 | 26% | 5 | 28% |  |  | 3 | 22% |  |  |
| WTp5 |  |  | 6 | 47% |  |  | 8 | 44% | 3 | 34% |  |  | 3 | 10% |  |  |
| WTp10 |  |  |  |  |  |  | 2 | 57% | 2 | 13% |  |  |  |  |  |  |

^a^ nucleotide change of TTT🡪CTT; ^b^ nucleotide change of TTT🡪TTG; ^c^ total number of mice out of n=8 (n=3 for WTp10) with variant at ≥ 5%; ^d^ average relative frequency of mutation in samples with mutation.
