# Supplementary material for: Obesity-Related Microenvironment Promotes Emergence of Virulent Influenza Virus Strains
Source: mBio. 2020 Mar 3;11(2):e03341-19. doi: 10.1128/mBio.03341-19 (PMC7064783; doi:10.1128/mBio.03341-19)
Supplement: TABLE S3 [file mBio.03341-19-st003.docx]

**Supplementary Table 3. Amino acid variants in OB- and WT-passaged viruses found at 5% relative frequency or more.**

| Protein | Nucleotide Position | Mutation | Amino Acid Annotation | p0 | OBp1 | | OBp5 | | WTp1 | | WTp5 | | | WTp10 | |
| --- | --- | --- | --- | --- | --- | --- | --- | --- | --- | --- | --- | --- | --- | --- | --- |
|  |  |  |  |  | %^a^ | #^b^ | % | # | % | # | % | | # | % | # |
| PB2 | 77 | A→G | D26G (GAC→GGC) | - | - | - | 8% | 1 | - | - | | - | - | - | - |
|  | 90 | A→G | I30M (ATA→ATG) | - | - | - | 6% | 1 | - | - | | 18% | 3 | - | - |
|  | 150 | G→A | M50I (ATG→ATA) | - | 11% | 1 | - | - | - | - | | - | - | - | - |
|  | 445 | C→T | P149S (CCT→TCT) | - | - | - | 6% | 1 | - | - | | - | - | - | - |
|  | 460 | C→A | L154I (CTC→ATC) | - | - | - | 19% | 3 | - | - | | - | - | - | - |
|  | 473 | A→G | E158G (GAG→GGG) | - | 100% | 1 | - | - | - | - | | - | - | - | - |
|  | 604 | A→T | M202L (ATG→TTG) | - | - | - | - | - | - | - | | 6% | 1 | - | - |
|  | 619 | C→A | L207I (CTA→ATA) | - | 17% | 1 | - | - | - | - | | - | - | - | - |
|  | 874 | G→A | V292I (GTA→ATA) | - | 11% | 2 | - | - | - | - | | - | - | - | - |
|  | 1,306 | A→G | R436G (AGG→GGG) | - | - | - | - | - | - | - | | 22% | 1 | - | - |
|  | 1,329 | A→C | K443N (AAA→AAC) | - | 33% | 2 | - | - | - | - | | - | - | - | - |
|  | 1,445 | A→G | K482R (AAA→AGA) | - | - | - | 52% | 3 | 13% | 2 | | 50% | 3 | - | - |
|  | 1,763 | C→T | T588I (ACC→ATC) | - | 15% | 1 | - | - | - | - | | - | - | - | - |
|  | 1,957 | T→C | S653P (TCT→CCT) | - | - | - | 17% | 1 | - | - | | - | - | - | - |
|  | 2,273 | T→C | I758T (ATC→ACC) | - | - | - | - | - | - | - | | 12% | 1 | - | - |
| PB1 | 33 | A→C | K11N (AAA→AAC) | - | - | - | - | - | - | - | | 9% | 1 | - | - |
|  | 114 | C→G | Y38* (TAC→TAG) | - | 12% | 1 | - | - | - | - | | - | - | - | - |
|  | 116 | C→G | T39S (ACC→AGC) | - | 12% | 1 | - | - | - | - | | - | - | - | - |
|  | 117 | C→G | T39T (ACC→ACG) | - | 11% | 1 | - | - | - | - | | - | - | - | - |
|  | 118 | A→C | M40L (ATG→CTG) | - | 11% | 1 | - | - | - | - | | - | - | - | - |
|  | 119 | T→C | M40T (ATG→ACG) | - | 10% | 1 | - | - | - | - | | - | - | - | - |
|  | 120 | G→T | M40I (ATG→ATT) | - | 10% | 1 | - | - | - | - | | - | - | - | - |
|  | 121 | G→A | D41N (GAC→AAC) | - | 10% | 1 | - | - | - | - | | - | - | - | - |
|  | 122 | A→T | D41V (GAC→GTC) | - | 10% | 1 | - | - | - | - | | - | - | - | - |
|  | 124 | A→T | T42S (ACA→TCA) | - | 10% | 1 | - | - | - | - | | - | - | - | - |
|  | 125 | C→G | T42R (ACA→AGA) | - | 12% | 1 | - | - | - | - | | - | - | - | - |
|  | 128 | T→G | V43G (GTA→GGA) | - | 9% | 1 | - | - | - | - | | - | - | - | - |
|  | 130 | A→C | N44H (AAC→CAC) | - | 11% | 1 | - | - | - | - | | - | - | - | - |
|  | 133 | A→T | R45* (AGA→TGA) | - | 9% | 1 | - | - | - | - | | - | - | - | - |
|  | 135 | A→C | R45S (AGA→AGC) | - | 9% | 1 | - | - | - | - | | - | - | - | - |
|  | 136 | A→C | T46P (ACA→CCA) | - | 9% | 1 | - | - | - | - | | - | - | - | - |
|  | 137 | C→G | T46R (ACA→AGA) | - | 9% | 1 | - | - | - | - | | - | - | - | - |
|  | 139 | C→T | H47Y (CAC→TAC) | - | 9% | 1 | - | - | - | - | | - | - | - | - |
|  | 140 | A→T | H47L (CAC→CTC) | - | 9% | 1 | - | - | - | - | | - | - | - | - |
|  | 143 | A→G | Q48R (CAA→CGA) | - | - | - | - | - | - | - | | 19% | 1 | - | - |
|  | 170 | C→T | T57I (ACA→ATA) | - | 7% | 1 | - | - | 7% | 1 | | - | - | - | - |
|  | 196 | C→T | L66F (CTC→TTC) | - | - | - | - | - | - | - | | - | - | 8% | 1 |
|  | 235 | C→T | P79S (CCA→TCA) | - | - | - | - | - | 13% | 1 | | - | - | - | - |
|  | 1,017 | G→T | M339I (ATG→ATT) | - | - | - | 12% | 1 | - | - | | - | - | - | - |
|  | 1,335 | C→T | D445D (GAC→GAT) | - | - | - | - | - | 7% | 1 | | - | - | - | - |
|  | 1,450 | A→G | I484V (ATA→GTA) | - | - | - | - | - | - | - | | 13% | 1 | - | - |
|  | 1,714 | A→G | R572G (AGA→GGA) | - | - | - | - | - | - | - | | 7% | 1 | - | - |
|  | 1,729 | A→G | K577E (AAG→GAG) | - | - | - | 19% | 1 | - | - | | - | - | - | - |
|  | 1,748 | C→T | T583I (ACC→ATC) | - | 10% | 1 | - | - | - | - | | - | - | - | - |
|  | 1,778 | A→G | D593G (GAT→GGT) | - | - | - | - | - | 35% | 1 | | - | - | - | - |
|  | 1,828 | T→C | C610R (TGC→CGC) | - | - | - | 6% | 1 | - | - | | - | - | - | - |
|  | 1,903 | A→G | K635E (AAA→GAA) | - | - | - | - | - | - | - | | - | - | - | - |
|  | 1,922 | A→G | N641S (AAC→AGC) | - | - | - | - | - | - | - | | 8% | 1 | - | - |
|  | 2,023 | C→A | L675I (CTC→ATC) | - | - | - | - | - | - | - | | 5% | 1 | - | - |
|  | 2,067 | C→A | Y689* (TAC→TAA) | - | - | - | - | - | 15% | 1 | | - | - | - | - |
|  | 2,072 | A→C | K691T (AAG→ACG) | - | - | - | - | - | 14% | 1 | | - | - | - | - |
|  | 2,074 | T→C | C692R (TGC→CGC) | - | - | - | - | - | 15% | 1 | | - | - | - | - |
|  | 2,075 | G→T | C692F (TGC→TTC) | - | - | - | - | - | 15% | 1 | | - | - | - | - |
|  | 2,076 | C→A | C692* (TGC→TGA) | - | - | - | - | - | 15% | 1 | | - | - | - | - |
|  | 2,083 | C→G | L695V (CTA→GTA) | - | - | - | - | - | 15% | 1 | | - | - | - | - |
|  | 2,084 | T→A | L695Q (CTA→CAA) | - | - | - | - | - | 15% | 1 | | - | - | - | - |
|  | 2,088 | C→G | F696L (TTC→TTG) | - | - | - | - | - | 15% | 1 | | - | - | - | - |
|  | 2,095 | T→C | F699L (TTT→CTT) | - | - | - | - | - | 15% | 1 | | - | - | - | - |
|  | 2,096 | T→A | F699Y (TTT→TAT) | - | - | - | - | - | 15% | 1 | | - | - | - | - |
|  | 2,097 | T→A | F699L (TTT→TTA) | - | - | - | - | - | 15% | 1 | | - | - | - | - |
|  | 2,099 | T→A | F700Y (TTC→TAC) | - | - | - | - | - | 15% | 1 | | - | - | - | - |
|  | 2,101 | C→A | P701T (CCT→ACT) | - | - | - | - | - | 15% | 1 | | - | - | - | - |
|  | 2,102 | C→A | P701H (CCT→CAT) | - | - | - | - | - | 15% | 1 | | - | - | - | - |
|  | 2,107 | A→G | S703G (AGT→GGT) | - | - | - | - | - | 14% | 1 | | - | - | - | - |
|  | 2,164 | G→A | A722T (GCC→ACC) | - | 27% | 2 | - | - | - | - | | - | - | - | - |
|  | 2,249 | T→A | I750N (ATT→AAT) | - | - | - | 6% | 1 | - | - | | - | - | - | - |
| PA | 940 | T→C | F314L (TTC→CTC) | - | - | - | - | - | 5% | 1 | | - | - | - | - |
|  | 1,045 | G→A | E349K (GAA→AAA) | - | - | - | 7% | 3 | - | - | | - | - | - | - |
|  | 1,154 | A→G | K385R (AAA→AGA) | - | 9% | 1 | - | - | - | - | | - | - | - | - |
|  | 1,255 | G→T | D419Y (GAT→TAT) | - | - | - | 8% | 1 | - | - | | - | - | - | - |
|  | 1,294 | G→A | V432I (GTT→ATT) | - | - | - | 33% | 1 | - | - | | - | - | - | - |
|  | 1,358 | G→A | C453Y (TGC→TAC) | - | - | - | 6% | 1 | - | - | | - | - | - | - |
|  | 1,472 | C→T | T491I (ACC→ATC) | - | - | - | - | - | - | - | | 7% | 1 | - | - |
|  | 1,473 | C→T | T491T (ACC→ACT) | - | 8% | 1 | - | - | - | - | | - | - | - | - |
|  | 1,496 | A→G | N499S (AAC→AGC) | - | - | - | - | - | - | - | | 100% | 1 | - | - |
|  | 1,497 | C→A | N499K (AAC→AAA) | - | - | - | - | - | - | - | | - | - | 6% | 1 |
|  | 1,595 | T→C | L532P (CTG→CCG) | - | - | - | 5% | 1 | - | - | | - | - | - | - |
|  | 1,874 | C→T | P625L (CCC→CTC) | - | 8% | 1 | - | - | - | - | | - | - | - | - |
|  | 1,913 | G→A | R638K (AGG→AAG) | - | - | - | 5% | 1 | - | - | | - | - | - | - |
|  | 2,008 | C→T | Q670* (CAG→TAG) | - | - | - | 14% | 1 | - | - | | - | - | - | - |
|  | 2,045 | A→G | D682G (GAT→GGT) | - | - | - | - | - | 9% | 1 | | - | - | - | - |
|  | 2,146 | A→G | K716E (AAG→GAG) | - | - | - | 6% | 1 | - | - | | - | - | - | - |
| PA-X | 103 | T→C | F35L (TTT→CTT) | - | 68% | 2 | 21% | 5 | - | - | | 38% | 5 | 6% | 1 |
|  | 105 | T→G | F35L (TTT→TTG) | - | 28% | 2 | 11% | 4 | 25% | 1 | | 35% | 2 | - | - |
|  | 121 | C→T | H41Y (CAT→TAT) | - | - | - | - | - | - | - | | 10% | 1 | - | - |
|  | 221 | A→G | H74R (CAC→CGC) | - | - | - | 14% | 1 | - | - | | - | - | - | - |
|  | 224 | G→A | R75Q (CGA→CAA) | - | - | - | 14% | 1 | - | - | | - | - | - | - |
|  | 227 | T→C | F76S (TTT→TCT) | - | - | - | 9% | 1 | - | - | | - | - | - | - |
|  | 228 | T→G | F76L (TTT→TTG) | - | - | - | 9% | 1 | - | - | | - | - | - | - |
|  | 298 | G→A | V100I (GTA→ATA) | - | - | - | - | - | - | - | | 16% | 3 | - | - |
| HA | 122 | T→C | V41A (GTA→GCA) | - | - | - | - | - | 12% | 1 | | - | - | - | - |
|  | 194 | C→T | A65V (GCC→GTC) | - | - | - | - | - | - | - | | 100% | 1 | - | - |
|  | 610 | G→A | D204N (GAC→AAC) | - | 45% | 1 | - | - | - | - | | - | - | - | - |
|  | 717 | T→C | ?239? (GNT→GNC) | - | - | - | - | - | 11% | 1 | | - | - | - | - |
|  | 1,265 | A→G | E422G (GAG→GGG) | - | 6% | 1 | - | - | - | - | | - | - | - | - |
|  | 1,642 | G→A | G548R (GGG→AGG) | - | - | - | - | - | 14% | 1 | | - | - | - | - |
| NP | 289 | T→G | Y97D (TAT→GAT) | - | - | - | - | - | 9% | 1 | | - | - | - | - |
|  | 342 | A→G | ?114? (GNA→GNG) | - | - | - | - | - | - | - | | 8% | 2 | - | - |
|  | 979 | C→T | Q327* (CAA→TAA) | - | - | - | - | - | 9% | 1 | | - | - | - | - |
|  | 1,136 | T→C | L379P (CTG→CCG) | - | - | - | - | - | - | - | | - | - | - | - |
|  | 1,349 | G→T | S450I (AGT→ATT) | - | - | - | - | - | - | - | | 8% | 1 | - | - |
|  | 1,364 | A→G | D455G (GAT→GGT) | - | - | - | - | - | 5% | 1 | | - | - | - | - |
|  | 1,456 | T→G | S486A (TCT→GCT) | - | - | - | - | - | - | - | | 5% | 2 | - | - |
| NA | 45 | G→T | M15I (ATG→ATT) | - | - | - | - | - | 7% | 1 | | - | - | - | - |
|  | 233 | A→G | Q78R (CAG→CGG) | - | - | - | - | - | - | - | | 6% | 2 | - | - |
|  | 248 | T→G | V83G (GTG→GGG) | - | - | - | - | - | - | - | | 9% | 2 | - | - |
|  | 276 | C→T | C92C (TGC→TGT) | - | - | - | - | - | - | - | | 7% | 1 | - | - |
|  | 670 | T→A | L224M (TTG→ATG) | - | - | - | 13% | 1 | - | - | | - | - | - | - |
|  | 1,007 | G→A | G336D (GGT→GAT) | - | - | - | 18% | 3 | - | - | | - | - | - | - |
|  | 1,323 | C→A | S441R (AGC→AGA) | - | 6% | 1 | - | - | - | - | | - | - | - | - |
| M1 | 136 | C→T | L46L (CTA→TTA) | - | - | - | - | - | 31% | 1 | | - | - | - | - |
|  | 281 | A→G | D94G (GAT→GGT) | - | - | - | - | - | 33% | 1 | | - | - | - | - |
|  | 427 | G→A | A143T (GCT→ACT) | - | - | - | - | - | 19% | 1 | | - | - | - | - |
|  | 515 | T→C | L172P (CTA→CCA) | - | - | - | - | - | - | - | | 17% | 1 | - | - |
| NS1 | 64 | T→C | F22L (TTT→CTT) | - | - | - | - | - | 25% | 1 | | - | - | - | - |
|  | 98 | T→C | L33P (CTT→CCT) | - | - | - | - | - | 12% | 1 | | - | - | - | - |
|  | 106 | C→A | L36I (CTC→ATC) | - | - | - | 7% | 1 | 45% | 1 | | 18% | 1 | - | - |
|  | 127 | T→C | L43L (TTA→CTA) | - | - | - | - | - | 8% | 1 | | - | - | - | - |
|  | 184 | A→G | K62E (AAA→GAA) | - | - | - | - | - | - | - | | 9% | 1 | - | - |
|  | 208 | A→G | K70E (AAA→GAA) | - | - | - | - | - | - | - | | 8% | 2 | - | - |
|  | 220 | A→G | S74G (AGC→GGC) | - | - | - | - | - | 12% | 1 | | - | - | - | - |
|  | 230 | T→C | L77P (CTT→CCT) | - | - | - | - | - | - | - | | - | - | - | - |
|  | 232 | A→G | R78G (AGA→GGA) | - | - | - | - | - | - | - | | - | - | - | - |
|  | 242 | T→C | I81T (ATT→ACT) | - | - | - | - | - | - | - | | - | - | - | - |
|  | 269 | T→C | L90P (CTT→CCT) | - | - | - | - | - | 9% | 1 | | - | - | - | - |
|  | 316 | A→G | M106V (ATG→GTG) | - | 14% | 3 | - | - | - | - | | 10% | 1 | - | - |
|  | 392 | A→G | K131R (AAA→AGA) | - | - | - | - | - | 10% | 1 | | - | - | - | - |
|  | 547 | G→A | G183R (GGA→AGA) | - | - | - | - | - | 6% | 1 | | - | - | - | - |
|  | 605 | C→T | A202V (GCT→GTT) | - | - | - | 21% | 3 | - | - | | 8% | 2 | 8% | 1 |
|  | 632 | G→A | R211K (AGA→AAA) | - | - | - | 6% | 2 | - | - | | - | - | - | - |
|  | 644 | C→A | P215H (CCT→CAT) | - | 29% | 3 | - | - | - | - | | - | - | - | - |
|  | 655 | A→G | K219E (AAA→GAA) | - | - | - | - | - | 13% | 1 | | - | - | - | - |
| NEP | 721 | G→A | M83I (ATG→ATA) | - | - | - | - | - | - | - | | - | - | 5% | 1 |
|  | 734 | A→G | K88E (AAA→GAA) | - | - | - | - | - | 27% | 1 | | - | - | - | - |
|  | 807 | A→G | E112G (GAG→GGG) | - | - | - | - | - | 13% | 1 | | - | - | - | - |
|  | 809 | A→G | I113V (ATA→GTA) | - | - | - | - | - | - | - | | 6% | 1 | - | - |

^a^ total number of mice out of n=3-5 with variant at ≥ 5%; ^b^ average relative frequency of mutation in samples with mutation. Mutations tagged with an asterisk (*) indicate stop codon and an interrogation mark (?) indicates unknown amino acid.
