# Supplementary material for: Obesity-Related Microenvironment Promotes Emergence of Virulent Influenza Virus Strains
Source: mBio. 2020 Mar 3;11(2):e03341-19. doi: 10.1128/mBio.03341-19 (PMC7064783; doi:10.1128/mBio.03341-19)
Supplement: TABLE S4 [file mBio.03341-19-st004.docx]

**Supplementary Table 4. Amino acid variation in DIO- and LN-passaged viruses found at 5% relative frequency or more.**

| Protein | Nucleotide Position | Mutation | Amino Acid Notation | p0 | DIOp1 | | | LNp1 | | DIOp4 | | | LNp4 | | |
| --- | --- | --- | --- | --- | --- | --- | --- | --- | --- | --- | --- | --- | --- | --- | --- |
|  |  |  |  |  | %^a^ | #^b^ | % | | # | | % | # | | % | # |
| PB2 | 56 | T→C | I19T (ATA→ACA) |  | 16% | 1 | - | | - | | - | - | | - | - |
|  | 98 | A→G | K33R (AAG→AGG) | - | 11% | 1 | - | | - | | - | - | | - | - |
|  | 473 | A→G | E158G (GAG→GGG) | - | - | - | - | | - | | 100% | 2 | | - | - |
|  | 1419 | G→A | M473I (ATG→ATA) | - | - | - | - | | - | | 10% | 1 | | - | - |
|  | 2000 | T→C | V667A (GTT→GCT) | - | - | - | - | | - | | 50% | 1 | | - | - |
| PB1 | 767 | A→G | E256G (GAA→GGA) | - | 11% | 1 | - | | - | | - | - | | - | - |
|  | 1210 | A→G | S404G (AGT→GGT) | - | 23% | 1 | - | | - | | - | - | | - | - |
|  | 2188 | T→G | F730V (TTC→GTC) | - | 6% | 1 | - | | - | | - | - | | - | - |
| PA | 1009 | G→T | A337S (GCT→TCT) | - | - | - | - | | - | | - | - | | 11% | 1 |
|  | 1048 | A→C | N350H (AAT→CAT) | - | - | - | 9% | | 1 | | - | - | | - | - |
|  | 1323 | G→A | M441I (ATG→ATA) | - | - | - | 5% | | 1 | | - | - | | - | - |
|  | 1666 | C→A | Q556K (CAA→AAA) | - | - | - | - | | - | | - | - | | 48% | 3 |
| PA-X | 343 | A→G | N115D (AAC→GAC) | - | - | - | - | | - | | - | - | | 13% | 1 |
| HA | 975 | A→T | K325N (AAA→AAT) | - | - | - | - | | - | | 93% | 1 | | - | - |
|  | 1298 | T→C | L433P (CTG→CCG) | - | 6% | 1 | - | | - | | - | - | | - | - |
|  | 1505 | A→G | D502G (GAC→GGC) | - | 12% | 1 | - | | - | | - | - | | - | - |
|  | 1507 | T→A | Y503N (TAC→AAC) | - | - | - | - | | - | | 23% | 1 | | - | - |
|  | 1510 | C→T | P504S (CCA→TCA) | - | - | - | - | | - | | 23% | 1 | | - | - |
|  | 1514 | A→C | K505T (AAA→ACA) | - | - | - | - | | - | | 23% | 1 | | - | - |
|  | 1516 | T→G | Y506D (TAC→GAC) | - | - | - | - | | - | | 23% | 1 | | - | - |
|  | 1519 | T→A | S507T (TCA→ACA) | - | - | - | - | | - | | 24% | 1 | | - | - |
|  | 1523 | A→T | E508V (GAG→GTG) | - | - | - | - | | - | | 24% | 1 | | - | - |
|  | 1527 | A→C | E509D (GAA→GAC) | - | - | - | - | | - | | 25% | 1 | | - | - |
|  | 1528 | G→A | A510T (GCA→ACA) | - | - | - | - | | - | | 25% | 1 | | - | - |
|  | 1602 | T→A | Y534* (TAT→TAA) | - | - | - | - | | - | | 31% | 1 | | - | - |
| NP | 130 | T→C | C44R (TGC→CGC) | - | - | - | - | | - | | 6% | 1 | | - | - |
|  | 660 | A→C | E220D (GAA→GAC) | - | 52% | 1 | - | | - | | - | - | | - | - |
|  | 733 | A→G | S245G (AGT→GGT) | - | - | - | - | | - | | 20% | 1 | | - | - |
|  | 1183 | A→G | N395D (AAT→GAT) | - | - | - | - | | - | | 25% | 1 | | - | - |
|  | 1252 | C→T | L418F (CTC→TTC) | - | - | - | - | | - | | 32% | 1 | | - | - |
|  | 1394 | A→G | E465G (GAG→GGG) | - | - | - | - | | - | | 29% | 1 | | - | - |
| NA | 469 | A→G | T157A (ACC→GCC) | - | - | - | - | | - | | 65% | 1 | | - | - |
|  | 727 | A→G | T243A (ACC→GCC) | - | - | - | - | | - | | 9% | 1 | | - | - |
|  | 754 | T→C | S252P (TCA→CCA) | - | - | - | - | | - | | 16% | 1 | | - | - |
|  | 1018 | T→C | S340P (TCT→CCT) | - | - | - | - | | - | | 6% | 1 | | - | - |
| M1 | 129 | G→A | M43I (ATG→ATA) | - | - | - | - | | - | | 6% | 1 | | - | - |
|  | 235 | T→C | F79L (TTT→CTT) | - | - | - | - | | - | | 7% | 1 | | - | - |
| M2 | 897 | A→G | E70G (GAG→GGG) | - | 7% | 1 | - | | - | | - | - | | - | - |
|  | 942 | A→G | D85G (GAT→GGT) | - | 7% | 1 | - | | - | | - | - | | - | - |
|  | 966 | A→G | N93S (AAC→AGC) | - | - | - | - | | - | | 9% | 1 | | - | - |
| NS1 | 122 | A→G | K41R (AAG→AGG) | - | 5% | 1 | - | | - | | - | - | | - | - |
|  | 159 | T→G | D53E (GAT→GAG) | - | 6% | 1 | - | | - | | - | - | | - | - |
|  | 194 | T→C | V65A (GTG→GCG) | - | - |  | - | | - | | 5% | 1 | | - | - |
|  | 208 | A→G | K70E (AAA→GAA) | - | 7% | 1 | - | | - | | - | - | | - | - |
|  | 238 | A→G | T80A (ACA→GCA) | - | - | - | - | | - | | 5% | 1 | | - | - |
|  | 314 | T→C | L105P (CTC→CCC) | - | 1% | 1 | - | | - | | - | - | | - | - |
|  | 326 | A→G | Q109R (CAA→CGA) | - | 6% | 1 | - | | - | | - | - | | - | - |
|  | 457 | G→A | E153K (GAG→AAG) | - | - | - | - | | - | | 7% | 1 | | - | - |
|  | 532 | G→A | V178I (GTT→ATT) | - | - | - | - | | - | | - | - | | 9% | 1 |
|  | 635 | C→T | P212L (CCT→CTT) | - | - | - | - | | - | | - | - | | 37% | 3 |
| NEP | 5 | A→C | D2A (GAC→GCC) | - | - | - | - | | - | | 6% | 1 | | - | - |
|  | 821 | T→C | S117P (TCG→CCG) | - | - | - | - | | - | | 10% | 1 | | - | - |

^a^ total number of mice out of n=3-5 with variant at ≥ 5%; ^b^ average relative frequency of mutation in samples with mutation. Mutations tagged with an asterisk (*) indicate stop codon and an interrogation mark (?) indicates unknown amino acid.
