# Supplementary material for: Obesity-Related Microenvironment Promotes Emergence of Virulent Influenza Virus Strains
Source: mBio. 2020 Mar 3;11(2):e03341-19. doi: 10.1128/mBio.03341-19 (PMC7064783; doi:10.1128/mBio.03341-19)
Supplement: TABLE S5 [file mBio.03341-19-st005.docx]

**Supplementary Table 5. NHBE cells used in study.**

| Donor | Race | Age | Gender | BMI^a^ |
| --- | --- | --- | --- | --- |
| 619261^b^ | White | 53 | Male | 38 |
| 613375 | Black | 65 | Female | 35 |
| 646466 | White | 38 | Male | 32 |
| 470899 | Hispanic | 37 | Male | 28 |
| 548314^b^ | White | 52 | Male | 25 |
| 619260 | White | 65 | Female | 20 |
| 628080 | Black | 42 | Male | 19 |

^a^ Body mass index (BMI). BMI ranges are underweight>18.5, normal=18.5 to 25.0, overweight >25.0 to 30.0, and obese ≥30.0. ^b^ donors used for age, race, and sex-matched comparisons.
